# Supplementary material for: Role of public involvement in the Royal College of Physicians’ Future Hospitals healthcare improvement programme: an evaluation
Source: BMJ Open. 2019 Sep 12;9(9):e027680. doi: 10.1136/bmjopen-2018-027680 (PMC6747633; doi:10.1136/bmjopen-2018-027680)
Supplement: Author's manuscript [file bmjopen-2018-027680.draft_revisions.pdf]

BMJ Open is committed to open peer review. As part of this commitment we make the peer review history of every article we publish publicly available.

When an article is published we post the peer reviewers' comments and the authors' responses online. We also post the versions of the paper that were used during peer review. These are the versions that the peer review comments apply to.

The versions of the paper that follow are the versions that were submitted during the peer review process. They are not the versions of record or the final published versions. They should not be cited or distributed as the published version of this manuscript.

BMJ Open is an open access journal and the full, final, typeset and author-corrected version of record of the manuscript is available on our site with no access controls, subscription charges or pay-per-view fees (http://bmjopen.bmj.com).

If you have any questions on BMJ Open's open peer review process please email info.bmjopen@bmj.com

BMJ Open

The role of public involvement in the Royal College of Physicians' Future Hospitals healthcare improvement programme

Journal:	*BMJ Open*
Manuscript ID	bmjopen-2018-027680
Article Type:	Research
Date Submitted by the Author:	13-Nov-2018
Complete List of Authors:	Frith, Lucy; University of Liverpool, Health Services Research<br>Hepworth, Lauren; University of Liverpool, Health Services Research<br>Lowers, Victoria; University of Liverpool, Health Services Research, Institute of Psychology, Health and Society<br>Joseph, Frank; Countess of Chester Hospital<br>Davies, Elizabeth; Royal College of Physicians<br>Gabbay, Mark
Keywords:	public involvement, Quality improvement, Royal College of Physicians, Future Hospital Programme, Theoretical evaluation

SCHOLARONE™
Manuscripts

The role of public involvement in the Royal College of Physicians' Future Hospitals healthcare improvement programme

Lucy Frith[1], Lauren Hepworth[1], Mark Gabbay[1], Victoria Lowers[1], Frank Joseph[2], Elizabeth Davies[2]

[1] University of Liverpool, Liverpool, UK

[2] Royal College of Physicians, London, UK

Address for correspondence:

Dr Lucy Frith

Department of Health Services Research

Waterhouse Building, Block B, First Floor,

University of Liverpool,

1-5 Brownlow Street,

Liverpool, L69 3GL

E: l.j.frith@liverpool.ac.uk

T: 0151 7955333

Word Count: 4,512

Key words: Patient public involvement, Future Hospital Programme, quality improvement, healthcare improvement, theoretical evaluation

For peer review only

Abstract

Objectives

The Royal College of Physician's Future Hospital Programme (FHP) set out a blueprint for a radical new model of care that put patient experience centre stage. It was a multi-faceted programme that acted as a support mechanism for sites to improve front-line services.

This paper will consider the role of public patient involvement (PPI) as a HCI measure in the Future Hospital Programme. It will explore the underlying theoretical assumptions for embedding PPI, how PPI contributed to the development sites projects (theoretical explanations of the mechanisms of change) and how PPI input was assessed and measured.

Setting

Secondary care. The FHP consisted of eight clinician-led healthcare improvement (HCI) hospital development sites with two phases over three years.

Participants

Development site clinical teams, patient representatives, the RCP's Patient and Carer Network, members of the FHP team and fellows and members of the RCP.

Design/Methods

This analysis is based on an independent evaluation of the FHP. A mixed methods approach was employed combining documentary analysis, qualitative interviews and a web-based survey.

Results

A form of 'ex-post theory', a programme theory of PPI as an improvement intervention in the FHP, was developed. The main aims of the PPI initiatives were to develop more patient-centred care and improve the patient experience. The mechanisms that were designed to meet these aims were: 1) Co-production of the development site's projects; 2) A better understanding of patient experience; and 3) Evaluation of patient experience.

For peer review only

Conclusion

The structure of the programme over two phases enabled learning from Phase I to be incorporated into Phase II. Having the PPI representatives closely involved in the development site projects; developing communities of practice; and the oversight and measuring activities, all acted as 'disciplinary structures' that embedded PPI and kept the patient experience at the forefront of the improvement initiatives.

Strengths and limitations of this study

- Contributes to theoretical developments in public involvement as a quality improvement measure.
- Develops an 'ex-post' theory of how public involvement can be used to improve front-line services.
- This evaluation was unable to get robust data on the effects of the public involvement activities on the development site projects.
- Points to areas for future development such as developing mechanisms for measuring the impact of public involvement on service improvement initiatives.

Background

The Future Hospital Programme (FHP) arose out of the Future Hospital Commission (FHC), established by the Royal College of Physicians (RCP) in 2012. The FHC was set up in a response to the RCP's *Hospitals on the Edge Report*[1], that found: 'A health system ill-equipped to cope with the needs of an aging population with increasingly complex clinical, care and support needs; hospitals struggling to cope with an increase in clinical demand; and a systematic failure to deliver coordinated, patient-centred care'[1]. The FHC aimed to, 'set out a radical new model of care' that put patient experience centre stage, 'Patient experience must

For peer review only - http://bmjopen.bmj.com/site/about/guidelines.xhtml

For peer review only

be valued as much as clinical effectiveness, and patients must be involved in service design and delivery'[2].

To take the recommendations and vision of the FHC into practice, the RCP set up the FHP in 2013. The RCP advertised for clinician-led healthcare improvement (HCI) proposals to become FHP development sites. The first phase was recruited in 2014 and focussed on improving care for older and frail patients. The second phase was a targeted call on integrated care and began in 2016. Applicants were required to:

- 'provide details of their projects and how these aligned with the principles of the FHC;
- demonstrate involvement of patient representatives in design and implementation;
- have a local, board level executive sponsor'[3].

The FHP was a multi-faceted programme of work that acted as a support mechanism for the sites to improve front-line services, but it did not provide any additional 'transformational' funding. There were four main ways that the FHP supported HCI in the development sites:

Public patient involvement (PPI) - one of the main elements of the FHP was to foster and support PPI in the development sites HCI projects. The aim was to ensure that the patient perspective was present throughout the projects and patients were actively involved in design and project implementation.

FHP support structures – an RCP FHP team was created with Future Hospital Officers appointed who provided clinical leadership support. Collaborative learning structures were developed, such as: learning events where site teams met and shared experiences; training in improvement methodologies; peer support; and creating a community of interest (the Future Hospital Partners Network)[3]. The RCP's Patient and Carer Network (PCN) (established in 2004 to give greater patient and carer input into the work of the RCP) also provided support to local lay representatives and peer learning opportunities across all sites.

For peer review only - http://bmjopen.bmj.com/site/about/guidelines.xhtml

Reporting processes – development sites were required to report regularly to the FHP team (both written reports and telephone calls). This ensured that the sites maintained momentum and received regular feedback.

Data collection and analysis – sites were given sessions on data analysis, project management and support for collecting and interpreting performance metrics and patient experience data. There was a, 'focus on measuring the true impact of clinically-led improvement or change…[to] enable clinical teams to improve patient-centred care and outcomes'[3].

This paper will consider the role of PPI as a HCI measure in the FHP. It will focus on how the FHP used PPI to drive change and improve patient experience, rather than on the content of these changes and the individual site's initiatives (these are reported elsewhere)[3, 9]. This reflects the aims of our evaluation [4], which was to evaluate the FHP as a programme of HCI, not the specific site improvement projects. This paper will explore the underlying theoretical assumptions for embedding PPI in the FHP, how the PPI work contributed to the development sites projects (theoretical explanations of the mechanisms of change) and how PPI input was assessed and measured. There is a growing literature on the role of PPI in HCI [5-8], but this still remains underexplored compared to the use of PPI in other areas, and this paper contributes to this developing area.

METHODS

We used a mixed methods approach that combined documentary analysis, qualitative interviews and a web-based survey. We collected data from: development site teams, the development site patient representatives and the RCP's Patient and Carer Network (PCN), personnel from the RCP both directly and indirectly involved with the programme and

For peer review only - http://bmjopen.bmj.com/site/about/guidelines.xhtml

members and fellows of the RCP. We also drew on material from the FHP documentation and individual site reports. Members of the team also attended some of the national meetings for the development sites. The data and analysis is available in the evaluation report[4], evaluations of the individual site projects [9], and the final FHP Report[3].

Four hundred and seventy-one documents from the development sites were reviewed, including: monthly, quarterly and annual reports, patient reports, learning event presentations, posters, feedback, monthly call notes, notes from site visits and personal communications. Focus groups were conducted with all site teams, which included the clinical lead and at least one patient representative. A web-based questionnaire was circulated to the development site team members including the patient representatives.

Focus groups were conducted with all the development site teams and a further focus group was conducted with seven local patient representatives and two PCN representatives, in which seven of the eight development sites were represented. One-to-one interviews were offered to all local patient, four were conducted via telephone and one patient representative provided a written account. One-to-one interviews were conducted with key personnel in the RCP, 17 individuals either face to face or via telephone, six from within the FHP core team and 11 who were indirectly involved with the FHP, including three senior members (see table 1 for an overview).

Table 1: Overview of qualitative data

	**Number**	**Participants**
**Focus group**	1	9 (7 – site patient representatives, 2 – PCN members)
	8	With each development site team<br>Between two and five team members.<br>All groups included the clinical lead and 5 out of the 8 groups included at least one patient representative.
**Interviews**	5	Patient representatives
	6	FHP core team
	11	Members of RCP (including 3 senior members)

For peer review only - http://bmjopen.bmj.com/site/about/guidelines.xhtml

All focus groups and interviews were recorded with consent and transcribed verbatim. A constant comparative thematic analysis was used to code the data [10]. Descriptive quantitative statistics were used to analyse the multiple-choice responses of the survey. To maintain participants' anonymity, development sites are referred to as DS1 to DS8, patient representatives as PR1 to PR6 and the personnel in the Future Hospital Programme team as RCP1 to RCP17 (see Table 2 for information on the participants who we quoted). Ethical approval was given by the evaluation team's Institution. The manuscript was prepared using the Standards for Quality Improvement Reporting Excellence (SQUIRE 2.0) [11].

Table 2: Participant information for those quoted in the paper

ID	Phase	Site	Role
PR1	1	5	Patient representative
PR2	1	5	Patient representative
PR3	1	4	Patient representative
PR4	1	3	Patient representative
PR5	2	6	Patient representative
PR6	2	7	Patient representative
DS3	1	3	Clinician
DS8	2	8	Clinician
RCP1	-	-	FHP Team
RCP4	-	-	FHP Team
RCP5	-	-	Other RCP employee
RCP6	-	-	FHP Team
RCP12	-	-	FHP Team

RESULTS

The FHC's statement that 'patient experience is as important as clinical effectiveness' underpinned the FHP's attempt to re-orientate hospital provision to be more responsive to patients' needs rather than organised around professional and organisational imperatives. his reflects long-standing drives to make healthcare more patient-centred [12], and the FHP aimed to do this by not only consulting with patients and the public but initiating processes and structures to facilitate greater co-production of the development sites HCI projects.

For peer review only - http://bmjopen.bmj.com/site/about/guidelines.xhtml

As has often been noted in the literature [13], HCI programmes seldom start with an explicit programme theory, defined as, 'an explanation of why the effects observed in a program are likely to have occurred' [14]. Simply describing interventions does not always ensure that they can be replicated or scaled up in other contexts. How certain outcomes come about, the underpinning social processes and the mechanisms that produce these outcomes need theoretical interpretation to aid replication of the intervention[15]. Developing theory can also form the basis of 'theory orientated evaluation', that aims to identify 'the rationale and assumptions about mechanisms that link programs' processes and inputs to outcomes (both intended and unintended)'[14]. In order to conduct a form of theory orientated evaluation, we developed a programme theory of PPI as a HCI intervention in the FHP. As the FHP did not set out an explicit programme theory at the outset, from the data used in the evaluation, we constructed a form of 'ex post theory' [14]. The main aims of the PPI procedures were to develop more patient-centred care and improve the patient experience. The mechanisms that were designed to meet these aims can be broken down into three elements:

1. Co-production of the initiatives – having PPI representatives closely involved in the development site teams will keep them focussed on patient experience and patient-centred care. Therefore, it will be less likely to be overlooked or marginalised.
2. A better understanding of patient experience – organisations and professionals may not know what constitutes 'good patient experience'. PPI can provide important, hitherto, missing information and perspectives on patient experience that can be fed into service redesign alongside operational and clinical information.
3. Evaluating patient experience – using patient experience data as a key metric for evaluating quality of care will re-orientate clinical teams to the importance of patient experience and aid the prioritisation of patient-centred care.

How these three mechanisms were operationalised in practice, what worked, and what other mechanisms came into play as the programme developed, are important for assessing how PPI functioned in the FHP and what lessons can be learned for future HCI projects. The results

For peer review only

will be organised under headings corresponding to the three mechanisms designed to meet the aims of PPI in the FHP.

Co-production of initiatives

Having PPI representatives closely involved in the development site teams was seen as a mechanism to begin to co-produce services and ensure the continual prioritisation of patient-centred care. PPI was implemented from the start of the FHP. The RCP's PCN was involved in the FHC and setting up the FHP. Each development site had at least one local PPI representative within their team and in, Phase II, sites had to show that they had a PPI strategy in place before being chosen. The PPI representatives were meant to be an integral part of the FHP team and be active participants in team meetings and decision-making processes. The majority (63.6%) of respondents to the development site survey reported that their project was partially co-produced with patients and 27.3% reported full co-production.

Although in each phase the development sites had the same remit and operating instructions it was clear that PPI did not happen in the same way and to the same degree in each site. Comparing the evidence across site reports and phases, there were different levels of involvement and the timings and format of PPI varied. In some instances the involvement was reported to be rather tokenistic and the patient representative's chance to influence service redesign was seen as limited (Table 3, quote 1).

In some of the sites, the PPI representative had little input into setting up the project or its design, but were more involved at later stages. Some participants felt they were brought in after the main decisions were taken (Table 3, quote 2 and 3). However, in some sites patient representatives were more actively involved in setting up and co-producing the projects from their inception (Table 3, quote 4).

For peer review only - http://bmjopen.bmj.com/site/about/guidelines.xhtml

The FHP central team reported that from their perspective, PPI was well embedded in the FHP and this enabled a more genuine form of co-production (Table 3, quote 5).

Table 3: Co-production of initiatives

Co-production of initiatives	
Quote 1	"Well I have done very little. I have, I haven't had no idea what the patient rep was supposed to do. In my opinion I was merely a tick in a box that said you have to have a patient rep." (PR4)
Quote 2	"It quite frankly is that you don't start off with asking the patients what they want, you start off usually with some enthusiastic usually a clinician, who has an idea about how things might be done better…. and the patients are asked to contribute to the development of that idea." (PR5)
Quote 3	"All you ever do is ask them to review what you have done rather than to input into it and you know there are these things where you go, hmm, this is not a co-production the patient is not at the heart of the process of the project." (PR6)
Quote 4	"[I was] very involved. We meet monthly with the … team which is an opportunity to share and discuss ongoing proposals and ideas or implementation of new approaches to working….The patient reps are treated with courtesy and respect and views are listened to and taken on board. We are considered to be an integral and vitally important part of the team." (PR2)
Quote 5	"unique to Future Hospital, in comparison to the other programmes of work. So there is lots of 'oh a patient was involved' tick type activity, throughout the College [RCP], and I think the difference particularly with the phase two sites is that there is proper co-production with the patients I hope that they feel that way, it certainly seems at least a big step along the route to co-production, than anything else that I have been involved with or seen or heard about so far." (RCP1)

A better understanding of patient experience

The FHP sought to get a better understanding of the patient experience in two main ways. First, the involvement of the PPI representatives in the site teams and second, the capture of wider patient experience data in each development site. These will be discussed in turn.

The involvement of the PPI representatives in the site teams were seen as important by members of the central FHP team as a way of keeping the orientation of the team on patient experience (Table 4, quote 1).

For peer review only - http://bmjopen.bmj.com/site/about/guidelines.xhtml

However, the role of the PPI representative, what it should be and who was an appropriate person to occupy it was an area that was debated by all FHP participants and there was little consensus on this. As one site noted: 'those that are able to meaningfully contribute to system redesign are not necessarily representative of the wider population'[3]. Underpinning these concerns was ambiguity over the concept and purpose of being a 'representative' and what 'representativeness' meant in practice (Table 4, quote 2 & 3).

Table 4: A better understanding of patient experience

A better understanding of patient experience	
Quote 1	"It has helped to keep the clinicians grounded, it has helped to keep the focus on patient experience." (RCP12)
Quote 2	"I am very much of the opinion that individual patients cannot represent patients as a whole unless it is very strange or peculiar circumstances. Probably, leaders of some patient organisation or something but even then it is a pretty poor sample." (PR5)
Quote 3	"There is still quite a lot of uncertainty about what your [PPI representative] role is. Are you giving a viewpoint as a patient who has experienced that service, so if you like common sense from an individual point of view or are you in a representative role are you trying to reflect a broader view of patients let's say who are acutely ill going in through a particular hospital. And, that I think hasn't been worked out nationally we haven't really got a sort of sense of what the, what the major aspects of a patient representative role are." (RCP12)
Quote 4	"I think that the disappointing thing is that one considers patient experience, to be reflected by a patient representative. Because patient experience is so much more than just one person coming in and saying…I think the word representative is a very difficult word because I am not sure that [our patient representative] could truly represent patients other than having been one…we have 70,000 patients a year, so the question in my mind is, you know if we are trying to extrapolate a representation of their experience, then having one person who has their own carried experience representing them is difficult." (DS3)
Quote 5	"If you have a patient representative the… are they a representative of the wider population or are they just bringing their own baggage to it, that is a big question and it is a big, you almost have to train people not to bring their baggage to the table and that is not easy." (RCP4)
Quote 6	"Patients will speak more openly with us [PPI representatives] than perhaps they feel they can do with the medical or other members of the team." (PR2) and without their input, "I don't believe their true voice would have been heard. And so some assumptions would have been made as to what the patient needs." (PR2)

For peer review only - http://bmjopen.bmj.com/site/about/guidelines.xhtml

A clinician in a development site, while supportive of having patients involved, questioned the utility of having a specific PPI representative, as they felt this just reflected one individual's opinion (Table 4, quote 4). There was a concern over the lack of capacity in PPI and one member of the central FHP team felt that training was needed to enable PPI representatives to step outside of their own concerns and be able to represent the wider group (Table 4, quote 5).

A second way of incorporating data on patient experience in the FHP was the systematic capture of patient experience data in each development site. Local patient and PCN representatives played a key role in organising and developing patient experience surveys and other types of data gathering such as focus groups or co-production events. The PPI representatives were involved in recruiting, training and supporting volunteers to pilot the patient experience surveys, provide feedback, propose improvements and develop site-specific tailored questionnaires [16]. These were seen as one of the successes of the FHP, that they were able to get the feedback from the wider body of patients and in a more sustained and nuanced way. One development site patient representative spoke about how they were able to get much richer and valuable data on patient experience and what patients wanted from services (see Table 4, quote 6).

Metrics on patient experience

An important part of the FHP was building capacity in data collection and its management so that they could demonstrate the impact of their service changes. One of the key elements of this was developing expertise in capturing, measuring and interpreting patient experience data. 'This metrics orientation provides focus and develops a language of 'measures' that 'quantifies' patient care' [3]. It was seen as important that the metrics captured reflected what was important to patients[3]. The role of this continual data collection in prioritising PPI was seen as crucial to the success of instituting PPI in the FHP (Table 5, quote 1).

For peer review only - http://bmjopen.bmj.com/site/about/guidelines.xhtml

290 **Table 5:** Metrics on patient experience

Metrics on patient experience	
Quote 1	"…the patient engagement piece is quite labour intensive, and whether without the Future Hospital Programme guiding it … will providers prioritise it [patient engagement] in the same way they would without the Future Hospital chasing them and asking them what they are doing on a regular basis...". (DS8)
Quote 2	"For phase two having watched how the sort of organic coming together, people volunteering option in phase one for patient reps, hadn't really been as effective as I think we initially hoped and that, the rigours of doing FHP alongside the day jobs…. meant that patient recruitment was often low down on their list and sometimes it took them, a good few months to recruit a patient rep…. So for phase two we decided to do a more proactive recruitment campaign advertised….. So we learnt a lot from phase one, about how to take patient involvement and engagement in the development site teams from sort of tokenistic and leaving the teams to do it themselves, to really prescribing what we needed and to get the framework in place to." (RCP6)

291

292 One of the benefits of this continual reporting and assessment of progress and the 293 incorporation of a form of the 'Plan-Do-Study-Act' (PDSA) cycle [17], was that the FHP's 294 approach to PPI evolved between the two phases. This was an iterative process where 295 lessons were learnt and improvements made as the programme progressed. Due to problems 296 experienced in Phase I, where some sites lacked any patient representation for periods of 297 time or had recruited individuals who had difficulties in contributing to the role, changes were 298 made to the application process to be a development site. In Phase II, 'Patient representatives 299 were identified in the application and were integral to the interview processes'[3]. The PCN 300 became involved in the selection of the sites and the recruitment for local site PPI 301 representatives became more formalised. The development of the PPI representatives' role 302 and the overall strategy and implementation over the two phases of the FHP is outlined by a 303 member of the FHP team (see Table 5, quote 2). Other improvements included providing more 304 support for the development site PPI representatives. For example, they were given a buddy 305 from the central PCN and this relationship became more formalised in Phase II.

306

307

For peer review only - http://bmjopen.bmj.com/site/about/guidelines.xhtml

DISCUSSION

The FHP commitment to involving local patients, PPI representatives and PCN members in service design, monitoring and evaluation was a new experience for the RCP and the PCN [16], and it was recognised by all parties that ‘doing’ PPI was challenging and required significant commitment on the part of all members of the development site teams.

The lack of underpinning theory can make evaluation challenging [13, 14]. In order to design, carry out and continuously evaluate PPI in HCI projects, having a working programme theory can provide clear aims and objectives against which to assess if and how PPI as an intervention has worked. The FHP did not include an impact assessment of the PPI activity itself and this is not surprising, as ways to measure the impact of PPI are generally under developed [18]. We developed an ‘ex-post programme theory’ of PPI as an HCI intervention and used this to assess whether the PPI mechanisms used in the FHP had fulfilled their aims.

The first mechanism was co-production of development site projects. The initial requirements for sites to have a PPI strategy, a patient representative and link with the PCN were important framing mechanisms for the project. This kept the focus on patient experience and it was by being a FHP development site that this was promoted and sustained. As site members observed, along with all the other requirements of both the HCI projects and day to day business, it would have been easy for PPI to get subsumed. The communities of learning that the FHP support structures created and attending learning events with other sites acted as a disciplinary structure, a community of practice that kept PPI at the forefront of the projects [14]. The support and endorsement for PPI from a prestigious medical college was also a key factor in PPI being seen as an important and worthwhile activity. This way of improving practice through professional communities and expert reassurance has been used in other studies, [19,] and worked well in the FHP, although other studies suggests more mixed evidence of their effectiveness [20].

For peer review only - http://bmjopen.bmj.com/site/about/guidelines.xhtml

The second mechanism was providing a better understanding of patient experience. A challenge with getting information on patient experience is that only a few patients were and could be involved in the PPI initiatives, and thus information could be argued to be partial. Even when sites tried to organise co-production days, these often did not always attract much interest from patients. Further, concepts of how representatives represent are contested in PPI [21]. Several sites saw the role of the patient representatives as a 'patient voice' rather than providing a representative account of 'patient experience'. Therefore, the ability to attract a range of patients' views and get wider understanding of patient experience was often limited. However, sites generally saw the value of including patient representatives in clinical teams and reported that they found the contribution of patients extremely valuable. Several sites were aiming to increase the level of patient input as a response to the high levels of changing demand among their population groups. Thus, adding extra in-house capacity and developing arrangements for closer working with patient groups was seen as a high priority for future service improvement. The work done by the patient representatives: running patient surveys, developing and piloting new iterations and exploring qualitative data gathering mechanisms, such as interviews and focus groups, helped produce much richer information and site teams used this to both monitor their progress and feed into further improvement cycles.

Finally, the FHP was designed to enable sites to instigate robust systems for measuring progress and giving the sites the skills to set up data gathering structures. 'Measurement always has consequences' [14] and by having patient experience data as a key performance indicator and the requirement to report to the central FHP team regularly, it ensured both that this remained an important focus for the projects, and that the patient representatives had a clearly defined and important role.

Learning between Phases

A strength of the FHP was employing learning from Phase I into Phase II and embedding a PDSA cycle in the programme. This meant key changes were made in Phase II that

For peer review only - http://bmjopen.bmj.com/site/about/guidelines.xhtml

strengthened PPI: involving the PCN in the process of choosing sites, strengthening processes for recruiting patient representatives, formalising the buddy system between the local representatives and a PCN member; and recommending each team have two patient representatives to share the work and provide support [16].

Limitations

In the evaluation of the FHP conducted by our team [4], we were not able to get detailed process or quantitative data on the activities undertaken by either the local or PCN patient representatives. As has been noted, assessing the impact of PPI on HCI projects is challenging, and embedding key measures of success throughout the project would have provided a useful way of assessing progress during the FHP.

CONCLUSIONS

The developing an ethos of co-production, even if this was often moving towards rather than fully achieving it, was seen as a major success of the FHP. To meaningfully embed PPI in HCI projects it needs to be sustained and sustainable, and requires significant investment in support structures, and time and space given so decisions can be genuinely co-produced. There needs to be support of the whole system – organisational buy in – so that it becomes a 'way of doing things' throughout the HCI project and once the initiative is rolled out. Demonstrating the impact of PPI in HCI projects is the next challenge and there is a need to develop methodologies that can capture relevant metrics and the nuances of involvement. Combining qualitative and quantitative data that are embedded throughout project can give a more multi-dimensional picture[22]. Attention to developing programme theories of PPI in HCI will be a key step and this paper has contributed to this debate by developing an 'ex-post theory' and used this to assess and evaluate the use of PPI in the FHP.

For peer review only - http://bmjopen.bmj.com/site/about/guidelines.xhtml

Acknowledgements

We would like to acknowledge and express thanks to all the members of the development site teams, the PCN, staff at the Royal College and Physicians and Members and Fellows of the RCP who took the time to speak to us or complete a survey.

The authors would also like to thank the Professor Tom Walley for his support with this work.

Professor Gabbay and Dr Frith are part funded by The National Institute for Health Research Collaboration for Leadership in Applied Health Research and Care North West Coast (NIHR CLAHRC NWC).

Disclaimer

The views expressed here are those of the authors and not necessarily those of the NHS, the NIHR, or the Department of Health and Social Care.

Funding statement

This evaluation was funded by the Royal College of Physicians.

Competing interests

We have not competing interests to declare.

Author contribution

LF – analysed the data, drafted the paper, LH, MG, VL – analysed the data, contributed to writing and revising the paper, FJ – contributed to writing and revising the paper, ED – public contributor, contributed to writing and revising the paper.

Data sharing statement

All requests should be directed to the corresponding author.

For peer review only

References

1. Royal College of Physicians. Hospitals on the edge? The time for action. London: RCP, 2012.

2. Future Hospital Commission. Future Hospital: Caring for medical patients. London: Royal College of Physicians, 2013.

3. Future Hospital Programme. Delivering the future hospital: full report London: Royal College of Physicians; 2017 [Available from: www.rcplondon.ac.uk/projects/outputs/future-hospital-programme-delivering-future-hospital.

4. Hepworth L, Antonacopoulou E, Frith L, et al. Future Hospital Programme: an independent evaluation, University of Liverpool, 2017. https://www.rcplondon.ac.uk/projects/outputs/future-hospital-programme-delivering-future-hospital (accessed 27/9/18)

5. Lavoie-Tremblay M, O'Connor P, Biron A, et al. The experience of patients engaged in co-designing care processes. The Health Care Manager 2016;35(4):284-93.

6. Renedo A, Marston CA, Spyridonidis D, et al. Patient and public involvement in healthcare quality improvement: how organizations can help patients and professionals to collaborate. Public Management Review 2015;17(1):17-34.

7. Fereday S, Rezel K. A guide to patient and public involvement in quality improvement. London: Healthcare Quality Improvement Partnership, 2017.

8. Armstrong N, Herbert G, Aveling EL, et al. Optimizing patient involvement in quality improvement. Health Expectations 2013;16(3):e36-e47.

9. Future Hospital development sites, RCP. https://www.rcplondon.ac.uk/projects/future-hospital-development-sites accessed 27/11/18

10. Braun V, Clarke V. Using thematic analysis in psychology. Qualitative Research in Psychology 2006;3(2):77-101.

11. Ogrinc G, et al. SQUIRE 2.0 (Standards for QUality Improvement Reporting Excellence) revised publication guidelines from a detailed consensus process BMJ Qual Saf 2015;0:1–7. doi:10.1136/bmjqs-2015-004411

For peer review only - http://bmjopen.bmj.com/site/about/guidelines.xhtml

12. Coulter A, Collins A. Making shared decision-making a reality: no decision about me, without me. London: The King's Fund, 2011.

13. Davidoff F, Dixon-Woods M, Leviton L, et al. Demystifying theory and its use in improvement. BMJ Quality and Safety 2015;24:228-38.

14. Dixon-Woods M, Bosk CL, Aveling EL, et al. Explaining Michigan: Developing an ex post theory of a quality improvement program. Milbank Quarterly 2011;89(2):167-205.

15. Howarth E, Devers K, Moore G, et al. Contextual issues and qualitative research. In Raine R, Fitzpatrick R, Barratt H, Bevan G, Black N, Boaden R, et al. Challenges, solutions and future directions in the evaluation of service innovations in health care and public health. Health Services Delivery Research 2016;4(16):105-20.

16. Caporn CA, Sawicka ZS. Making ripples: patient involvement in service planning. Future Hospital Journal 2016;3(2):142-46.

17. Institute for Healthcare Improvement. QI Essentials Toolkit: Plan-Do-Study-Act worksheet Cambridge, Massachusetts, USA Institute for Healthcare Improvement; 2017 [Available from: www.ihi.org/resources/Pages/Tools/PlanDoStudyActWorksheet.aspx.

18. Mockford C, Staniszewska S, Griffiths F, et al. The impact of patient and public involvement on UK NHS health care: a systematic review. International Journal for Quality in Health Care 2012;24(1):28-38.

19. Jeffs L, McShane J, Flintoft V, et al. Contextualizing learning to improve care using collaborative communities of practices. BMC Health Services Research 2016;16(464) doi: 10.1186/s12913-016-1566-4

20. Aveling E-L, Martin G, Armstrong N, et al. Quality improvement through clinical communities: Eight lessons for practice. Journal of Health Organisation and Management 2012;26(2):158-74.

21. Maguire K, Britten N. "How can anybody be representative for those kind of people?" Forms of patient representation in health research, and why it is always contestable. Social Science and Medicine 2017;183:62-69.

For peer review only

For peer review only - http://bmjopen.bmj.com/site/about/guidelines.xhtml

22. Raine R, Fitzpatrick R, Barratt H, et al. Challenges, solutions and future directions in the evaluation of service innovations in health care and public health. 4 2016;16

For peer review only

For peer review only - http://bmjopen.bmj.com/site/about/guidelines.xhtml

Research and reporting methodology
Revised **Standards for QUality Improvement Reporting Excellence** (**SQUIRE 2.0**) publication guidelines

Notes to authors

- The SQUIRE guidelines provide a framework for reporting new knowledge about how to improve healthcare.
- The SQUIRE guidelines are intended for reports that describe system level work to improve the quality, safety and value of healthcare, and used methods to establish that observed outcomes were due to the intervention(s).
- A range of approaches exists for improving healthcare. SQUIRE may be adapted for reporting any of these.
- Authors should consider every SQUIRE item, but it may be inappropriate or unnecessary to include every SQUIRE element in a particular manuscript.
- The SQUIRE glossary contains definitions of many of the key words in SQUIRE.
- The explanation and elaboration document provides specific examples of well-written SQUIRE items and an in-depth explanation of each item.
- Please cite SQUIRE when it is used to write a manuscript.

Text section and item name	Page/line no(s). info is located
**Title and abstract**	
1. **Title**	
Indicate that the manuscript concerns an initiative to improve healthcare (broadly defined to include the quality, safety, effectiveness, patient-centredness, timeliness, cost, efficiency and equity of healthcare).	Line 1
2. **Abstract**	
a. Provide adequate information to aid in searching and indexing.	Lines 4-24
b. Summarise all key information from various sections of the text using the abstract format of the intended publication or a structured summary such as: background, local problem, methods, interventions, results, conclusions.	Page 1, lines 4-30
**Introduction: Why did you start?**	
3. **Problem description** - Nature and significance of the local problem.	Lines 51-59
4. **Available knowledge** - Summary of what is currently known about the problem, including relevant previous studies.	Lines 103-105<br>Lines 149-154
5. **Rationale** - Informal or formal frameworks, models, concepts and/or theories used to explain the problem, any reasons or assumptions that were used to develop the intervention(s) and reasons why the intervention(s) was expected to work	Lines 69-71<br>Lines 163-172
6. **Specific aims** - Purpose of the project and of this report.	Lines 96-105
**Methods: What did you do?**	
7. **Context** - Contextual elements considered important at the outset of introducing the intervention(s).	Lines 51-71

For peer review only

8. **Intervention(s)**	
a. Description of the intervention(s) in sufficient detail that others could reproduce it.	Lines 71-94
b. Specifics of the team involved in the work.	Line 114 – reference to full evaluation report
9. **Study of the intervention(s)**	
a. Approach chosen for assessing the impact of the intervention(s).	Lines 108-109
b. Approach used to establish whether the observed outcomes were due to the intervention(s).	We were unable to do this due to the evaluation design lines 329-336
10. **Measures**	
a. Measures chosen for studying processes and outcomes of the intervention(s), including rationale for choosing them, their operational definitions and their validity and reliability.	Lines 108-115
b. Description of the approach to the ongoing assessment of contextual elements that contributed to the success, failure, efficiency and cost.	Partly N/A as this was an evaluation of a programme that had partly taken place<br>Lines 117-130
c. Methods employed for assessing completeness and accuracy of data.	Line 114
11. **Analysis**	
a. Qualitative and quantitative methods used to draw inferences from the data.	Lines 108-115
b. Methods for understanding variation within the data, including the effects of time as a variable.	Lines 117-116
12. **Ethical considerations** - Ethical aspects of implementing and studying the intervention(s) and how they were addressed, including, but not limited to, formal ethics review and potential conflict(s) of interest.	Line 134-137
**Results: What did you find?**	
13. **Results**	
a. Initial steps of the intervention(s) and their evolution over time (eg, time-line diagram, flow chart or table), including modifications made to the intervention during the project.	Lines 61-71<br>Lines 251-264
b. Details of the process measures and outcomes.	We were unable to do this due to the evaluation design lines 329-336
c. Contextual elements that interacted with the intervention(s).	Lines 190-195
d. Observed associations between outcomes, interventions and relevant contextual elements.	Lines 243-249
e. Unintended consequences such as unexpected benefits, problems, failures or costs associated with the intervention(s).	Lines 254-264

For peer review only

f. Details about missing data.	Lines 329-336
**Discussion: What does it mean?**	
14. **Summary**	
a. Key findings, including relevance to the rationale and specific aims.	Lines 267-270
b. Particular strengths of the project.	Lines 277-279<br>Lines 279-292<br>Lines 302-311<br>Lines 321-326
15. **Interpretation**	
a. Nature of the association between the intervention(s) and the outcomes.	N/a
b. Comparison of results with findings from other publications.	Lines 287-292<br>Lines 298-299<br>Lines 313-318
c. Impact of the project on people and systems.	Lines 302-311
d. Reasons for any differences between observed and anticipated outcomes, including the influence of context.	N/A
e. Costs and strategic trade-offs, including opportunity costs.	N/A
16. **Limitations**	
a. Limits to the generalisability of the work.	Lines 329-336
b. Factors that might have limited internal validity such as confounding, bias or imprecision in the design, methods, measurement or analysis.	Lines 329-336
c. Efforts made to minimise and adjust for limitations.	N/A
**Conclusions**	
a. Usefulness of the work.	Lines 340-344
b. Sustainability.	Lines 303-307
c. Potential for spread to other contexts.	Lines 303-307
d. Implications for practice and for further study in the field.	Lines 345-346
e. Suggested next steps.	Lines 347-349
**Other information**	
18. **Funding** - Sources of funding that supported this work. Role, if any, of the funding organisation in the design, implementation, interpretation and reporting.	Included in the submission

Ogrinc G, et al. BMJ Qual Saf 2015;0:1–7. doi:10.1136/bmjqs-2015-004411
Downloaded from http://qualitysafety.bmj.com/ on January 2, 2017

For peer review only

BMJ Open

The role of public involvement in the Royal College of Physicians' Future Hospitals healthcare improvement programme: an evaluation.

Journal:	*BMJ Open*
Manuscript ID	bmjopen-2018-027680.R1
Article Type:	Research
Date Submitted by the Author:	15-May-2019
Complete List of Authors:	Frith, Lucy; University of Liverpool, Health Services Research<br>Hepworth, Lauren; University of Liverpool, Health Services Research<br>Lowers, Victoria; University of Liverpool, Health Services Research, Institute of Psychology, Health and Society<br>Joseph, Frank; Countess of Chester Hospital<br>Davies, Elizabeth; Royal College of Physicians<br>Gabbay, Mark
**Primary Subject Heading**:	Patient-centred medicine
Secondary Subject Heading:	Sociology
Keywords:	public involvement, Quality improvement, Royal College of Physicians, Future Hospital Programme, Theoretical evaluation, co-production

SCHOLARONE™
Manuscripts

The role of public involvement in the Royal College of Physicians' Future Hospitals healthcare improvement programme: an evaluation

Lucy Frith[1], Lauren Hepworth[1], Victoria Lowers[1], Frank Joseph[2], Elizabeth Davies[2], Mark Gabbay[1]

[1] University of Liverpool, Liverpool, UK

[2] Royal College of Physicians, London, UK

Address for correspondence:

Dr Lucy Frith

Department of Health Services Research

Waterhouse Building, Block B, First Floor,

University of Liverpool,

1-5 Brownlow Street,

Liverpool, L69 3GL

E: l.j.frith@liverpool.ac.uk

T: 0151 7955333

Word Count: 4,512

Key words: Patient public involvement, co-production, Future Hospital Programme, quality improvement, healthcare improvement, theoretical evaluation

For peer review only - http://bmjopen.bmj.com/site/about/guidelines.xhtml

Abstract

Objectives

The Royal College of Physician's Future Hospital Programme (FHP) set out a blueprint for a radical new model of care that put patient experience centre stage. This paper reports the results of an independent evaluation of the FHP and focusses on the role public patient involvement (PPI) played in these projects, how PPI was operationalised in this context and the benefits and challenges of moving towards co-production of improvement initiatives.

Setting

Secondary care. The FHP consisted of eight clinician-led healthcare improvement (HCI) hospital development sites with two phases over three years.

Participants

Development site clinical teams, patient representatives, the RCP's Patient and Carer Network, members of the FHP team and fellows and members of the RCP.

Design/Methods

This analysis is based on an independent evaluation of the FHP using FHP documentation, and data collected specifically for the evaluation: qualitative interviews, focus groups and a web-based survey.

Results

The main aims of the PPI initiatives were to develop more patient-centred care and improve the patient experience. The mechanisms designed to meet these aims were: 1) A programme of PPI in the development site's projects; 2) A better understanding of patient experience; and 3) Evaluation of patient experience.

Conclusion

This evaluation of the FHP identifies some key elements that need to be considered when attempting to more closely integrate PPI and co-production within service re-design. The structure of FHP over two phases enabled learning from Phase I to be incorporated into Phase II, including a greater emphasis on, and support for, PPI and co-production. Having the PPI

For peer review only - http://bmjopen.bmj.com/site/about/guidelines.xhtml

representatives closely involved in the development site projects; developing communities of practice; and the oversight and measuring activities, all acted as 'disciplinary structures' that contributed to embedding PPI in the FHP and kept the patient experience at the forefront of the improvement initiatives.

Strengths and limitations of this study

- Contributes to theoretical developments in public involvement as a quality improvement measure.
- Develops an 'ex-post' theory of how public involvement can be used to improve front-line services.
- This evaluation was unable to get robust data on the effects of the public involvement activities on the development site projects.
- Points to areas for future development such as developing mechanisms for measuring the impact of public involvement on service improvement initiatives.

Background

The Future Hospital Programme (FHP) arose out of the Future Hospital Commission (FHC), established by the Royal College of Physicians (RCP) in 2012. The FHC was set up in a response to the RCP's *Hospitals on the Edge Report*[1], that found: 'A health system ill-equipped to cope with the needs of an aging population with increasingly complex clinical, care and support needs; hospitals struggling to cope with an increase in clinical demand; and a systematic failure to deliver coordinated, patient-centred care'[1]. The FHC aimed to, 'set out a radical new model of care' that put patient experience centre stage, 'Patient experience must be valued as much as clinical effectiveness, and patients must be involved in service design and delivery'[2]. A key part of the FHC was to foster and promote patient-centred care, which they defined as, 'individualised, compassionate, holistic and collaborative… [and] continuous, care in settings appropriate to the patient's clinical and care needs viewed from their

For peer review only - http://bmjopen.bmj.com/site/about/guidelines.xhtml

perspective.'[2] While there is debate in the literature over how this concept is and should be defined,[3] key elements of patient-centred care for the FHC were: continuity of care, reduction in unnecessary moving of patients between wards and better integration with social care services post- discharge.

To take the recommendations and vision of the FHC into practice, the RCP set up the FHP as a 5-year programme in 2013, running until 2017. The RCP advertised for clinician-led healthcare improvement (HCI) proposals to become FHP development sites. The first phase was recruited in 2014 and focussed on improving care for older and frail patients. The second phase was a targeted call on integrated care and began in 2016. Each phase included four pilot project sites, all had to align their objectives with the Future Hospital Commission principles (full details of the FHP have been published elsewhere[4 5]). Applicants were required to:

- 'provide details of their projects and how these aligned with the principles of the FHC;
- demonstrate involvement of patient representatives in design and implementation;
- have a local, board level executive sponsor'[4].

The FHP was a multi-faceted programme of work that acted as a support mechanism for the sites to improve front-line services, but it did not provide any additional 'transformational' funding. There were four main ways that the FHP supported HCI in the development sites:

Public patient involvement (PPI) - one of the main elements of the FHP was to foster and support PPI and co-production in the development sites HCI projects working closely with the RCP Patient Carer Network (PCN) (established in 2004 to give greater patient and carer input into the work of the RCP). The aim was to have the patient perspective present throughout the projects and involve patient representatives actively in the co-production of the project design and implementation.

For peer review only - http://bmjopen.bmj.com/site/about/guidelines.xhtml

FHP support structures – an RCP FHP team was established with a senior physician appointed as Future Hospital Officer for each phase who provided clinical leadership support. Collaborative learning structures were developed, such as: learning events where site teams met and shared experiences; training in improvement methodologies; peer support; and creating a community of interest (the Future Hospital Partners Network)[4]. The RCP's PCN also provided support to local lay representatives and peer learning opportunities across all sites.

Reporting processes – development sites were required to report monthly to the FHP team (both written reports and telephone calls). This ensured that the sites maintained momentum and received regular feedback.

Data collection and analysis – sites were given training sessions provided by the RCP or external experts on data analysis, project management and support for collecting and interpreting performance metrics and patient experience data. There was a, 'focus on measuring the true impact of clinically-led improvement or change…[to] enable clinical teams to improve patient-centred care and outcomes'[4].

This paper reports the results of an independent evaluation of the FHP and focusses on the role public patient involvement (PPI) played. The aim of the paper is to explore the perceptions and experiences of the central FHP team, the patient representatives and the development site teams of how PPI was operationalised in this context and the benefits and challenges of this work.

The terms patient public involvement and co-production are contested and in defining these terms it is important to recognise that, in the messy realties of practice, how patients and the public participated in the FHP did not always conform to one definitional model, but often

For peer review only - http://bmjopen.bmj.com/site/about/guidelines.xhtml

included and straddled different types of practice: from passive involvement, such as being consulted about planned changes; to more active involvement, such as being involved in planning; and, in some cases, co-production of initiatives[4 5]. Tritter defines PPI as: 'Ways in which patients can draw on their experience and contributors of the public can apply their priorities to the evaluation, development, organization and delivery of health services.'[6] We will use INVOLVE's definition of co-production: 'Co-producing a research project is an approach in which researchers, practitioners and the public work together, sharing power and responsibility from the start to the end of the project, including the generation of knowledge.'[7] Definitions of healthcare improvement are also contested[8]. We will define HCI broadly as: 'better patient experience and outcomes achieved through changing provider behaviour and organisation through using a systematic change method and strategies.'[9]

There is a growing literature on the role of PPI in HCI [5-8], but this still remains underexplored compared to the use of PPI in other areas, and this paper contributes to this developing area. There is a large literature on theoretical considerations of topologies of involvement and a number of attempts to develop categorisations to capture what PPI is[6 10 11] and what an 'ideal type' might be[12]. Arnsteins' ladder of engagement is often quoted as one of the first examples of this, where the bottom rung is manipulation and the top rung is citizens' power [13]. This paper does not attempt to compare or measure PPI in the FHP according to abstract standards, but contributes to the empirical literature on the practice of PPI in service design and quality improvement. It has been noted that although there is a substantial body of theoretical literature, there is a gap on *how* involvement and co-design is carried out in practice[14]. It is widely recognised that the context of interventions are key[15] and this paper provides an example of a specific programme of PPI to add to the literature on exploring contextual factors and developing good practice in the area of PPI in HCI.

For peer review only - http://bmjopen.bmj.com/site/about/guidelines.xhtml

METHODS

This paper is based on a summative evaluation[16] that our team conducted of the FHP as a programme of HCI. It does not consider the not the specific site improvement projects nor content of these changes (these are reported elsewhere)[4, 17-24]. Our team became involved towards the end of the programme and we did not have input in the design of the projects, nor the procedures, content and timing for gathering metrics. This was an evaluation rather than a primary research project and this paper reports on the perceptions and experiences of the central FHP team, the patient representatives and the development site teams, of how PPI was operationalised in this context and the benefits and challenges of this work. Thus, this is a consideration of what happened in a HCI initiative in the real world, not in a structured research project. This has a number of strengths and limitations as a method. The strengths are that it provides information on what actually happened in practice, it demonstrates the workings of PPI embedded in the context of shifting priorities and changes in the NHS, such as funding and personnel changes. The main weakness is that from the data collected by the FHP and our evaluation it was difficult to ascertain the effects of the PPI initiatives on the individual development site projects.

We used a multi-source approach using a variety of data sources: analysis of the FHP documentation, central reporting, individual site progress reports, and records of meetings. We also collected data specifically for the evaluation: qualitative interviews, focus groups and a web-based survey. We conducted these with: the development site teams, the development site patient representatives and the RCP's PCN, personnel from the RCP both directly and indirectly involved with the programme and members and fellows of the RCP. Members of the team also attended some of the national meetings for the development sites. The data and analysis is available in the evaluation report[5], evaluations of the individual site projects[17-24] and the final FHP Report[4].

Four hundred and seventy-one documents from the development sites were reviewed, including: monthly, quarterly and annual reports, patient reports, learning event presentations, posters, feedback, monthly call notes, notes from site visits and personal communications. Focus groups were conducted with all the development site teams which included the clinical lead and at least one patient representative. A further focus group was conducted with seven local patient representatives and two PCN representatives, in which seven of the eight development sites were represented. One-to-one interviews were offered to all local patient representatives, four were conducted via telephone and one patient representative provided a written account. One-to-one interviews were conducted with key personnel in the RCP, 17 individuals either face to face or via telephone, six from within the FHP core team and 11 who were indirectly involved with the FHP, including three senior members (see table 1 for an overview).

A web-based questionnaire was circulated to the development site team members including the patient representatives after the focus groups, providing another opportunity for team members to share their opinions individually. The survey used a combination of multiple choice and free text response options, with questions based on the topic guide for the focus groups. The survey received 22 responses; this included at least one response from each of the eight development sites and from a mixture of patient representatives and clinicians (for details see [5]).

Table 1: Overview of qualitative data

	**Number**	**Participants**
**Focus group**	1	9 (7 – site patient representatives, 2 – PCN members)
	8	With each development site team<br>Between two and five team members.<br>All groups included the clinical lead and 5 out of the 8 groups included at least one patient representative.
**Interviews**	5	Patient representatives
	6	FHP core team
	11	Members of RCP (including 3 senior members)

For peer review only - http://bmjopen.bmj.com/site/about/guidelines.xhtml

All focus groups and interviews were recorded with consent and transcribed verbatim. A constant comparative thematic analysis was used to code the data[25]. Descriptive quantitative statistics were used to analyse the multiple-choice responses of the survey. To maintain participants' anonymity, development sites are referred to as DS1 to DS8, patient representatives as PR1 to PR6 and the personnel in the Future Hospital Programme team as RCP1 to RCP17 (see Table 2 for information on the participants who we quoted). Ethical approval was given by the evaluation team's Institution. The manuscript was prepared using the Standards for Quality Improvement Reporting Excellence (SQUIRE 2.0)[26].

ID	Phase	Site	Role
PR1	1	5	Patient representative
PR2	1	5	Patient representative
PR3	1	4	Patient representative
PR4	1	3	Patient representative
PR5	2	6	Patient representative
PR6	2	7	Patient representative
DS3	1	3	Clinician
DS8	2	8	Clinician
RCP1	-	-	FHP Team
RCP4	-	-	FHP Team
RCP5	-	-	Other RCP employee
RCP6	-	-	FHP Team
RCP12	-	-	FHP Team

Table 2: Participant information for those quoted in the paper

For peer review only - http://bmjopen.bmj.com/site/about/guidelines.xhtml

RESULTS

The FHC saw ‘patient experience as important as clinical effectiveness’ and that patient-centred care should drive the improvement of services. This commitment underpinned the FHP’s attempt to re-orientate hospital provision to be more responsive to patients’ needs rather than organised around professional and organisational imperatives. The FHP aimed to do this by not only consulting with patients and the public but initiating processes and structures to facilitate greater PPI and co-production in the development sites HCI projects.

As has often been noted in the literature[27], HCI programmes seldom start with an explicit programme theory, defined as, ‘an explanation of why the effects observed in a program are likely to have occurred’[28]. Simply describing interventions does not always ensure that they can be replicated or scaled up in other contexts. How certain outcomes come about, the underpinning social processes and the mechanisms that produce these outcomes need theoretical interpretation to aid replication of the intervention[29]. Developing theory can also form the basis of ‘theory orientated evaluation’, that aims to identify ‘the rationale and assumptions about mechanisms that link programs’ processes and inputs to outcomes (both intended and unintended)’[28]. In order to conduct a form of theory orientated evaluation, we developed a programme theory of PPI as a HCI intervention in the FHP. As the FHP did not set out an explicit programme theory at the outset, we constructed a form of ‘ex post theory’’[28]. This was developed from the data we gathered as part of the evaluation and the wider FHP documentation and constructed during the data analysis phase of our evaluation.

The main aims of the PPI procedures were to develop more patient-centred care and consequently improve the patient experience. The mechanisms that were designed to meet these aims can be broken down into three elements:

1. Greater PPI and co-production of the initiatives – having PPI representatives closely involved in the development site teams will keep them focussed on developing patient-

For peer review only - http://bmjopen.bmj.com/site/about/guidelines.xhtml

centred care and improving the patient experience, so that this is less likely to be overlooked or marginalised.

2. A better understanding of patient experience – organisations and professionals may not know what constitutes ‘good patient experience’. PPI can provide important, hitherto, missing information and perspectives on patient experience that can be fed into service redesign alongside operational and clinical information.
3. Evaluating patient experience – using patient experience data as a key metric for evaluating quality of care will re-orientate clinical teams to the importance of patient experience and aid the prioritisation of patient-centred care.

How these mechanisms were operationalised in practice and determining the successful aspects of the FHP, are important for thinking through how PPI can be used in service change and improvement and what lessons can be learned for future HCI projects.

PPI and co-production of initiatives

Having PPI representatives closely involved in the development site teams was seen as a mechanism to begin to involve patients and move towards greater co-production of services, to ensure the continual prioritisation of patient-centred care. PPI was implemented from the start of the FHP. The RCP’s PCN was involved in the FHC and setting up the FHP. Each development site had at least one local PPI representative within their team and in Phase II, sites had to show that they had a PPI strategy in place before being chosen. The PPI representatives were meant to be an integral part of the FHP team and be active participants in team meetings and decision-making processes. The majority (63.6%) of respondents to the development site web-based survey reported that their project was partially co-produced with patients and 27.3% reported full co-production. Co-production was limited in some sites, particularly in Phase I, where some projects were already fairly well-developed before any PPI took place.

For peer review only

Although in each phase the development sites had the same remit and operating instructions it was clear that PPI did not happen in the same way and to the same degree in each site. Comparing the evidence across site reports and phases, there were different levels of involvement and the timings and format of PPI varied. In some instances the involvement was reported to be rather ‘tokenistic’ and the patient representative’s chance to influence service redesign was seen as limited (Table 3, quote 1).

In some of the sites, the PPI representative had little input into setting up the project or its design, but were more involved at later stages. Some participants felt they were brought in after the main decisions were taken (Table 3, quote 2 and 3). However, in some sites patient representatives were more actively involved in setting up and co-producing the projects from their inception (Table 3, quote 4).

The FHP central team reported that from their perspective, PPI was well embedded in the FHP and this enabled more co-production of projects (Table 3, quote 5).

Table 3: Levels of involvement and co-production of initiatives

Involvement and co-production of initiatives	
Quote 1	“Well I have done very little. I have, I haven’t had no idea what the patient rep was supposed to do. In my opinion I was merely a tick in a box that said you have to have a patient rep.” (PR4)
Quote 2	“It quite frankly is that you don’t start off with asking the patients what they want, you start off usually with some enthusiastic usually a clinician, who has an idea about how things might be done better…. and the patients are asked to contribute to the development of that idea.” (PR5)
Quote 3	“All you ever do is ask them to review what you have done rather than to input into it and you know there are these things where you go, hmm, this is not a co-production the patient is not at the heart of the process of the project.” (PR6)
Quote 4	“[I was] very involved. We meet monthly with the … team which is an opportunity to share and discuss ongoing proposals and ideas or implementation of new approaches to working….The patient reps are treated with courtesy and respect and views are listened to and taken on board. We are considered to be an integral and vitally important part of the team.” (PR2)

For peer review only - http://bmjopen.bmj.com/site/about/guidelines.xhtml

Quote 5	"unique to Future Hospital, in comparison to the other programmes of work. So there is lots of 'oh a patient was involved' tick type activity, throughout the College [RCP], and I think the difference particularly with the phase two sites is that there is proper co-production with the patients I hope that they feel that way, it certainly seems at least a big step along the route to co-production, than anything else that I have been involved with or seen or heard about so far." (RCP1)

A better understanding of patient experience

The FHP sought to get a better understanding of patient experience in two main ways. First, the involvement of the PPI representatives in the site teams and second, the capture of wider patient experience data in each development site. These will be discussed in turn.

The involvement of the PPI representatives in the site teams were seen as important by members of the central FHP team as a way of keeping the focus of the development site teams on patient experience (Table 4, quote 1).

However, the role of the PPI representative, what it should be and who was an appropriate person to occupy this type of role was debated by all FHP participants and there was little consensus on this. The key area of debate was who could be said to accurately provide insights into the patient experience, how do we know we are not just getting a partial view, or views that may not be held by the majority of patients? As one site noted: 'those that are able to meaningfully contribute to system redesign are not necessarily representative of the wider population'[4]. Underpinning these concerns was ambiguity over the concept and purpose of being a 'representative' and what 'representativeness' meant in practice (Table 4, quote 2 & 3).

For peer review only - http://bmjopen.bmj.com/site/about/guidelines.xhtml

Table 4: A better understanding of patient experience

A better understanding of patient experience	
Quote 1	"It has helped to keep the clinicians grounded, it has helped to keep the focus on patient experience." (RCP12)
Quote 2	"I am very much of the opinion that individual patients cannot represent patients as a whole unless it is very strange or peculiar circumstances. Probably, leaders of some patient organisation or something but even then it is a pretty poor sample." (PR5)
Quote 3	"There is still quite a lot of uncertainty about what your [PPI representative] role is. Are you giving a viewpoint as a patient who has experienced that service, so if you like common sense from an individual point of view or are you in a representative role are you trying to reflect a broader view of patients let's say who are acutely ill going in through a particular hospital. And, that I think hasn't been worked out nationally we haven't really got a sort of sense of what the, what the major aspects of a patient representative role are." (RCP12)
Quote 4	"I think that the disappointing thing is that one considers patient experience, to be reflected by a patient representative. Because patient experience is so much more than just one person coming in and saying…I think the word representative is a very difficult word because I am not sure that [our patient representative] could truly represent patients other than having been one…we have 70,000 patients a year, so the question in my mind is, you know if we are trying to extrapolate a representation of their experience, then having one person who has their own carried experience representing them is difficult." (DS3)
Quote 5	"If you have a patient representative the… are they a representative of the wider population or are they just bringing their own baggage to it, that is a big question and it is a big, you almost have to train people not to bring their baggage to the table and that is not easy." (RCP4)
Quote 6	"Patients will speak more openly with us [PPI representatives] than perhaps they feel they can do with the medical or other members of the team." (PR2) and without their input, "I don't believe their true voice would have been heard. And so some assumptions would have been made as to what the patient needs." (PR2)

A clinician in a development site, while supportive of having patients involved, questioned the utility of having a specific PPI representative, as they felt this just reflected one individual's opinion (Table 4, quote 4). There was a concern over the lack of capacity in PPI and one member of the central FHP team felt that training was needed to enable PPI representatives to step outside of their own concerns and be able to represent the wider group (Table 4, quote 5).

For peer review only - http://bmjopen.bmj.com/site/about/guidelines.xhtml

A second way of incorporating data on patient experience in the FHP was the systematic capture of patient experience data in each development site.[30] Local patient and PCN representatives played a key role in organising and developing patient experience surveys and other types of data gathering such as running focus groups or co-production events. The PPI representatives were involved in recruiting, training and supporting volunteers to pilot the patient experience surveys, provide feedback, propose improvements and develop site-specific tailored questionnaires[30]. These were seen as one of the successes of the FHP, that they were able to get the feedback from the wider body of patients and in a more sustained and nuanced way. One development site patient representative spoke about how they were able to get much richer and valuable data on patient experience and what patients wanted from services (see Table 4, quote 6).

Metrics on patient experience

An important part of the FHP was building capacity in data collection and its management so that they could demonstrate the impact of their service changes. One of the key elements of this was developing expertise in capturing, measuring and interpreting patient experience data. 'This metrics orientation provides focus and develops a language of 'measures' that 'quantifies' patient care'[4]. It was seen as important that the metrics captured reflected what was important to patients[4]. The role of this continual data collection in prioritising PPI was seen as crucial to the success of instituting PPI in the FHP (Table 5, quote 1).

Table 5: Metrics on patient experience

Metrics on patient experience	
Quote 1	"…the patient engagement piece is quite labour intensive, and whether without the Future Hospital Programme guiding it … will providers prioritise it [patient engagement] in the same way they would without the Future Hospital chasing them and asking them what they are doing on a regular basis...". (DS8)
Quote 2	"For phase two having watched how the sort of organic coming together, people volunteering option in phase one for patient reps, hadn't really been as effective as I think we initially hoped and that, the

For peer review only - http://bmjopen.bmj.com/site/about/guidelines.xhtml

	rigours of doing FHP alongside the day jobs…. meant that patient recruitment was often low down on their list and sometimes it took them, a good few months to recruit a patient rep…. So for phase two we decided to do a more proactive recruitment campaign advertised….. So we learnt a lot from phase one, about how to take patient involvement and engagement in the development site teams from sort of tokenistic and leaving the teams to do it themselves, to really prescribing what we needed and to get the framework in place to." (RCP6)

One of the benefits of this continual reporting and assessment of progress and the incorporation of a form of the 'Plan-Do-Study-Act' (PDSA) cycle[31], was that the FHP's approach to PPI evolved between the two phases. This was an iterative process where lessons were learnt and improvements made as the programme progressed. Due to problems experienced in Phase I, where some sites lacked any patient representation for periods of time or had recruited individuals who had difficulties in contributing to the role, changes were made to the application process to be a development site. In Phase II, 'Patient representatives were identified in the application and were integral to the interview processes'[4]. The PCN became involved in the selection of the sites and the recruitment for local site PPI representatives became more formalised. The development of the PPI representatives' role and the overall strategy and implementation over the two phases of the FHP is outlined by a member of the FHP team (see Table 5, quote 2). Other improvements included providing more support for the development site PPI representatives. For example, they were given a buddy from the central PCN and this relationship became more formalised in Phase II.

DISCUSSION

The FHP commitment to involving local patients, PPI representatives and PCN members in service design, monitoring and evaluation was a new experience for the RCP and the PCN[30]. It was recognised by all parties that 'doing' PPI was challenging and required significant commitment on the part of all members of the development site teams.

The lack of theoretical consideration of how PPI might contribute to service change and create the conditions for patient-centred care can make evaluation challenging, and the paucity of

For peer review only - http://bmjopen.bmj.com/site/about/guidelines.xhtml

underpinning theory in this area has been noted in the literature[27 28]. In order to design, carry out and continuously evaluate PPI in HCI projects, having a working programme theory can provide clear aims and objectives against which to assess whether the PPI mechanisms have fulfilled their stated aims and if, and how, PPI as an intervention has worked. We developed an 'ex-post programme theory' of PPI as an HCI intervention. How such theoretical underpinnings of PPI as an intervention can and should be developed, are areas that needs further investigation. Our theory, by beginning to set out the aims and objectives of this kind of programme, contributes to this. More work needs to be done on how to assess whether the aims of such programmes are met and what are appropriate metrics for assessing PPI as a HCI tool.

The initial requirements for sites to have a PPI strategy, a patient representative and good links with the PCN were important framing mechanisms for the project. This kept the focus on patient experience and it was by being a FHP development site that this was promoted and sustained. As site members observed, along with all the other requirements of both the HCI projects and day-to-day business, it would have been easy for PPI to get subsumed. The communities of learning that the FHP support structures created and attending learning events with other sites[4] acted as a disciplinary structure, a community of practice that kept PPI at the forefront of the projects[28]. The support and endorsement for PPI from a prestigious medical college was also a key factor in PPI being seen as an important and worthwhile activity. This way of improving practice through professional communities and expert reassurance has been used in other studies[32], and worked well in the FHP, although other studies suggests more mixed evidence of their effectiveness[33].

A challenge with getting comprehensive information on patient experience is that only a few patients were and could be involved in the PPI initiatives, and therefore, this information could be argued to only partially capture the patient experience. Even when sites tried to organise co-production days, these often did not attract much interest from patients. Further, concepts

For peer review only

For peer review only - http://bmjopen.bmj.com/site/about/guidelines.xhtml

of how patient representatives 'represent' are contested in PPI[34]. Several sites saw the role of the patient representatives as a 'patient voice' rather than providing a representative account of 'patient experience'. Therefore, the ability to attract a range of patients' views and get wider understanding of patient experience was often limited. However, sites generally saw the value of including patient representatives in clinical teams and reported that they found the contribution of patients extremely valuable. Several sites were aiming to increase the level of patient input, to help them response to the high levels of changing demand among their population groups. Thus, adding extra in-house capacity and developing arrangements for closer working with patient groups was seen as a high priority for future service improvement. The work done by the patient representatives: running patient surveys, developing and piloting new iterations and exploring qualitative data gathering mechanisms, such as interviews and focus groups, helped produce much richer information and site teams used this to both monitor their progress and feed into further improvement cycles.

Finally, the FHP was designed to enable sites to instigate robust systems for measuring progress and giving the sites the skills to set up data gathering structures. 'Measurement always has consequences'[28] and by having patient experience data as a key performance indicator and the requirement to report to the central FHP team regularly, it ensured both that this remained an important focus for the projects, and that the patient representatives had a clearly defined and important role.

Learning between Phases

A strength of the FHP was employing learning from Phase I into Phase II and embedding a PDSA cycle in the programme. This meant key changes were made in Phase II that strengthened PPI: involving the PCN in the process of choosing sites, strengthening processes for recruiting patient representatives, formalising the buddy system between the local representatives and a PCN member; and recommending each team have two patient representatives to share the work and provide support[30].

For peer review only - http://bmjopen.bmj.com/site/about/guidelines.xhtml

Limitations

This was an evaluation[5] of an existing and ongoing project and the data and measurement processes were not determined by the evaluation team. Thus, this evaluation is based on the available data and resources. The FHP did not include an impact assessment of the PPI activity itself and hence, we were not able to get detailed process or quantitative data on the activities undertaken by either the local or PCN patient representatives[5]. As has been noted, assessing the impact of PPI on HCI projects is challenging, and embedding key measures of success throughout the project would have provided a useful way of assessing progress during the FHP. The lack of studies on how PPI effects the outcomes of service reconfigurations has been noted in Dalton et al's review[35], and ways to measure the impact of PPI are generally under-researched.[36] This is an area that needs further research to develop ways of effectively capturing the impact and effects of PPI in service design and change.

CONCLUSIONS

Developing an ethos of co-production, even if this was often moving towards rather than fully achieved, was seen as a major success of the FHP. To meaningfully embed PPI in HCI projects it needs to be sustained and sustainable, and requires significant investment in support structures, both centrally and locally, plus time and space allocated to enabling decisions to be genuinely co-produced. There needs the support of the whole system – organisational buy in – so that it becomes a 'way of doing things' throughout the HCI project and once the initiative is rolled out. Additionally, the project teams need dedicated time for meetings to start the design process, rather than involving patient representatives when the projects are already set up, as this inevitably precludes genuine co-production. This increases the investment of staff time and associated costs to deliver real PPI and co-production. Demonstrating the impact of PPI in HCI projects is the next challenge and there is a need to develop methodologies that can capture relevant metrics and the nuances of involvement. Combining qualitative and quantitative data and embedding collection throughout the project can give a more multi-dimensional picture[37]. Attention to developing programme theories of

For peer review only - http://bmjopen.bmj.com/site/about/guidelines.xhtml

PPI in HCI will be a key step and this paper aimed to contribute to this debate by developing an ‘ex-post theory’ to suggest ways of developing PPI as an HCI tool.

Acknowledgements

We would like to acknowledge and express thanks to all the members of the development site teams, the PCN, staff at the Royal College and Physicians and Members and Fellows of the RCP who took the time to speak to us or complete a survey.

The authors would also like to thank the Professor Tom Walley for his support with this work.

Professor Gabbay and Dr Frith are part funded by The National Institute for Health Research Collaboration for Leadership in Applied Health Research and Care North West Coast (NIHR CLAHRC NWC).

Disclaimer

The views expressed here are those of the authors and not necessarily those of the NHS, the NIHR, or the Department of Health and Social Care.

Funding statement

This evaluation was funded by the Royal College of Physicians.

Competing interests

We have not competing interests to declare.

Author contribution

LF – analysed the data, drafted the paper, LH, MG, VL – analysed the data, contributed to writing and revising the paper, FJ – contributed to writing and revising the paper, ED – public contributor, contributed to writing and revising the paper.

For peer review only

Data sharing statement

All requests should be directed to the corresponding author.

References

1. Royal College of Physicians. Hospitals on the edge? The time for action. London: RCP, 2012.
2. Future Hospital Commission. Future Hospital: Caring for medical patients. London: Royal College of Physicians, 2013.
3. Dewing J, McCormack B. Tell me, how do you define person-centredness? *Journal of Clinical Nursing* 2016;26(17-18):2509-10.
4. Future Hospital Programme. Delivering the future hospital: full report London: Royal College of Physicians; 2017 [Available from: www.rcplondon.ac.uk/projects/outputs/future-hospital-programme-delivering-future-hospital.
5. Hepworth L, Antonacopoulou E, Frith L, et al. Future Hospital Programme: an independent evaluation - Final Report: University of Liverpool, 2017.
6. Tritter JQ. Revolution or evolution: the challenges of conceptualizing patient and public involvement in a consumerist world. *Health Expectations* 2009;12(3):275-87.
7. INVOLVE. Guidance on co-producing a research project. Southampton: INVOLVE, 2018.
8. The Health Foundation. Quality improvement made simple: What everyone should know about health care quality improvement. London: The Health Foundation, 2013.
9. Øvretveit J. Does improving quality save money? A review of the evidence of which improvements to quality reduce costs to health service providers. London: The Health Foundation, 2009.
10. Oliver SR, Rees RW, Clarke-Jones L, et al. A multidimensional conceptual framework for analysing public involvement in health services research. *Health Expectations* 2008;11(1):72-84.
11. Rowe G, Frewer LJ. A typology of public engagement mechanisms. *Science, Technology and Human Values* 2005;30(2):251-90.
12. Fredriksson M, Tritter JQ. Disentangling patient and public involvement in healthcare decisions: why the difference matters. *Sociology of Health and Illness* 2017;39(1):95-111.
13. Arnstein SR. A ladder of citizen participation. *Journal of American Planning Association* 1969;35(4):216-24.
14. Ward ME, De Brún A, Beirne D, et al. Using co-design to develop a collective leadership intervention for healthcare teams to improve safety culture. *International Journal of Environmental Research and Public Health* 2018;15(6) doi: 10.3390/ijerph15061182
15. De Weger E, Van Vooren N, Luijkx KG, et al. Achieving successful community engagement: a rapid realist review. *BMC Health Services Research* 2018;18(285) doi: 10.1186/s12913-018-3090-1
16. The Health Foundation. Evaluation: what to consider. Commonly asked questions about how to approach evaluation of quality improvement in health care. London: The Health Foundation, 2015.
17. Williams O, Betsi Cadwaladr University Health Board Future Hospital Development Site Team. Phase 1 Future Hospital Development Site: Betsi Cadwaladr University Health Board Final Report. London: Royal College of Physicians, 2017.
18. East Lancashire Hospitals NHS Trust Future Hospital Development Site Team. Phase 1 Future Hospital Development Site: East Lancashire Hospitals NHS Trust Final Report. London: Royal College of Physicians, 2017.
19. Duckitt R, Hunt D, Finch L, et al. Phase 1 Future Hospital Development Site: Worthing Hospital Final Report. London: Royal College of Physicians, 2017.
20. Mid Yorkshire Hospitals NHS Trust Future Hospital Development Site Team. Phase 1 Future Hospital Development Site: Mid Yorkshire NHS Trust Final Report. London: Royal College of Physicians, 2017.

For peer review only - http://bmjopen.bmj.com/site/about/guidelines.xhtml

21. Kane B, O'Reilly S, Noble R, et al. Phase 2 Future Hospital Development Site: Central and South Manchester Final Report. London: RCP, 2017.

22. Arkwright P, Dixon S, Stafford N. Phase 2 Future Hospital Development Site: North West Paediatric Allergy Network. London: Royal College of Physicians, 2017.

23. Compton L, Lawn L, Wilkinson P, et al. Phase 2 Future Hospital Development Site: North West Surrey Final Report. London: Royal College of Physicians, 2017.

24. Rajasakeran A, Barlow R, Chadderton M, et al. Phase 2 Future Hospital Development Site: Sandwell and West Birmingham Final Report. London: Royal College of Physicians, 2017.

25. Braun V, Clarke V. Using thematic analysis in psychology. *Qualitative Research in Psychology* 2006;3(2):77-101.

26. Ogrinc G, Davies L, Goodman D, et al. SQUIRE 2.0 (Standards for QUality Improvment Reporting Excellence) revised publications guidelines from a detailed consesnus process. *BMJ Quality and Safety* 2016;25(12) doi: 10.1136/bmjqs-2015-004411

27. Davidoff F, Dixon-Woods M, Leviton L, et al. Demystifying theory and its use in improvement. *BMJ Quality and Safety* 2015;24:228-38.

28. Dixon-Woods M, Bosk CL, Aveling EL, et al. Explaining Michigan: Developing an ex post theory of a quality improvement program. *Milbank Quarterly* 2011;89(2):167-205.

29. Howarth E, Devers K, Moore G, et al. Contextual issues and qualitative research. In Raine R, Fitzpatrick R, Barratt H, Bevan G, Black N, Boaden R, *et al.* Challenges, solutions and future directions in the evaluation of service innovations in health care and public health. *Health Services Delivery Research* 2016;4(16):105-20.

30. Caporn CA, Sawicka ZS. Making ripples: patient involvement in service planning. *Future Hospital Journal* 2016;3(2):142-46.

31. Institute for Healthcare Improvement. QI Essentials Toolkit: Plan-Do-Study-Act worksheet Cambridge, Massachusetts, USA Institute for Healthcare Improvement; 2017 [Available from: www.ihi.org/resources/Pages/Tools/PlanDoStudyActWorksheet.aspx.

32. Jeffs L, McShane J, Flintoft V, et al. Contextualizing learning to improve care using collaborative communities of practices. *BMC Health Services Research* 2016;16(464) doi: 10.1186/s12913-016-1566-4

33. Aveling E-L, Martin G, Armstrong N, et al. Quality improvement through clinical communities: Eight lessons for practice. *Journal of Health Organisation and Management* 2012;26(2):158-74.

34. Maguire K, Britten N. "How can anybody be representative for those kind of people?" Forms of patient representation in health research, and why it is always contestable. *Social Science and Medicine* 2017;183:62-69.

35. Dalton J, Chambers D, Harden M, et al. Service user engagement and health service reconfiguration: a rapid evidence synthesis. *Health Services Delivery Research* 2015;3(17) doi: 10.3310/hsdr03170

36. Mockford C, Staniszewska S, Griffiths F, et al. The impact of patient and public involvement on UK NHS health care: a systematic review. *International Journal for Quality in Health Care* 2012;24(1):28-38.

37. Raine R, Fitzpatrick R, Barratt H, et al. Challenges, solutions and future directions in the evaluation of service innovations in health care and public health. *4* 2016;16

For peer review only - http://bmjopen.bmj.com/site/about/guidelines.xhtml

Research and reporting methodology

Revised **Standards for QUality Improvement Reporting Excellence** (**SQUIRE 2.0**) publication guidelines

Notes to authors

- The SQUIRE guidelines provide a framework for reporting new knowledge about how to improve healthcare.
- The SQUIRE guidelines are intended for reports that describe system level work to improve the quality, safety and value of healthcare, and used methods to establish that observed outcomes were due to the intervention(s).
- A range of approaches exists for improving healthcare. SQUIRE may be adapted for reporting any of these.
- Authors should consider every SQUIRE item, but it may be inappropriate or unnecessary to include every SQUIRE element in a particular manuscript.
- The SQUIRE glossary contains definitions of many of the key words in SQUIRE.
- The explanation and elaboration document provides specific examples of well-written SQUIRE items and an in-depth explanation of each item.
- Please cite SQUIRE when it is used to write a manuscript.

Text section and item name	Page/line no(s). info is located
**Title and abstract**	
1. **Title**	
Indicate that the manuscript concerns an initiative to improve healthcare (broadly defined to include the quality, safety, effectiveness, patient-centredness, timeliness, cost, efficiency and equity of healthcare).	Line 1
2. **Abstract**	
a. Provide adequate information to aid in searching and indexing.	Lines 4-24
b. Summarise all key information from various sections of the text using the abstract format of the intended publication or a structured summary such as: background, local problem, methods, interventions, results, conclusions.	Page 1, lines 4-30
**Introduction: Why did you start?**	
3. **Problem description** - Nature and significance of the local problem.	Lines 51-59
4. **Available knowledge** - Summary of what is currently known about the problem, including relevant previous studies.	Lines 103-105<br>Lines 149-154
5. **Rationale** - Informal or formal frameworks, models, concepts and/or theories used to explain the problem, any reasons or assumptions that were used to develop the intervention(s) and reasons why the intervention(s) was expected to work	Lines 69-71<br>Lines 163-172
6. **Specific aims** - Purpose of the project and of this report.	Lines 96-105
**Methods: What did you do?**	
7. **Context** - Contextual elements considered important at the outset of introducing the intervention(s).	Lines 51-71

For peer review only

8. **Intervention(s)**	
a. Description of the intervention(s) in sufficient detail that others could reproduce it.	Lines 71-94
b. Specifics of the team involved in the work.	Line 114 – reference to full evaluation report
9. **Study of the intervention(s)**	
a. Approach chosen for assessing the impact of the intervention(s).	Lines 108-109
b. Approach used to establish whether the observed outcomes were due to the intervention(s).	We were unable to do this due to the evaluation design lines 329-336
10. **Measures**	
a. Measures chosen for studying processes and outcomes of the intervention(s), including rationale for choosing them, their operational definitions and their validity and reliability.	Lines 108-115
b. Description of the approach to the ongoing assessment of contextual elements that contributed to the success, failure, efficiency and cost.	Partly N/A as this was an evaluation of a programme that had partly taken place<br>Lines 117-130
c. Methods employed for assessing completeness and accuracy of data.	Line 114
11. **Analysis**	
a. Qualitative and quantitative methods used to draw inferences from the data.	Lines 108-115
b. Methods for understanding variation within the data, including the effects of time as a variable.	Lines 117-116
12. **Ethical considerations** - Ethical aspects of implementing and studying the intervention(s) and how they were addressed, including, but not limited to, formal ethics review and potential conflict(s) of interest.	Line 134-137
**Results: What did you find?**	
13. **Results**	
a. Initial steps of the intervention(s) and their evolution over time (eg, time-line diagram, flow chart or table), including modifications made to the intervention during the project.	Lines 61-71<br>Lines 251-264
b. Details of the process measures and outcomes.	We were unable to do this due to the evaluation design lines 329-336
c. Contextual elements that interacted with the intervention(s).	Lines 190-195
d. Observed associations between outcomes, interventions and relevant contextual elements.	Lines 243-249
e. Unintended consequences such as unexpected benefits, problems, failures or costs associated with the intervention(s).	Lines 254-264

For peer review only

f. Details about missing data.	Lines 329-336
**Discussion: What does it mean?**	
14. **Summary**	
a. Key findings, including relevance to the rationale and specific aims.	Lines 267-270
b. Particular strengths of the project.	Lines 277-279<br>Lines 279-292<br>Lines 302-311<br>Lines 321-326
15. **Interpretation**	
a. Nature of the association between the intervention(s) and the outcomes.	N/a
b. Comparison of results with findings from other publications.	Lines 287-292<br>Lines 298-299<br>Lines 313-318
c. Impact of the project on people and systems.	Lines 302-311
d. Reasons for any differences between observed and anticipated outcomes, including the influence of context.	N/A
e. Costs and strategic trade-offs, including opportunity costs.	N/A
16. **Limitations**	
a. Limits to the generalisability of the work.	Lines 329-336
b. Factors that might have limited internal validity such as confounding, bias or imprecision in the design, methods, measurement or analysis.	Lines 329-336
c. Efforts made to minimise and adjust for limitations.	N/A
**Conclusions**	
a. Usefulness of the work.	Lines 340-344
b. Sustainability.	Lines 303-307
c. Potential for spread to other contexts.	Lines 303-307
d. Implications for practice and for further study in the field.	Lines 345-346
e. Suggested next steps.	Lines 347-349
**Other information**	
18. **Funding** - Sources of funding that supported this work. Role, if any, of the funding organisation in the design, implementation, interpretation and reporting.	Included in the submission

Ogrinc G, et al. BMJ Qual Saf 2015;0:1–7. doi:10.1136/bmjqs-2015-004411
Downloaded from http://qualitysafety.bmj.com/ on January 2, 2017

For peer review only

BMJ Open

The role of public involvement in the Royal College of Physicians' Future Hospitals healthcare improvement programme: an evaluation.

Journal:	*BMJ Open*
Manuscript ID	bmjopen-2018-027680.R2
Article Type:	Research
Date Submitted by the Author:	09-Jul-2019
Complete List of Authors:	Frith, Lucy; University of Liverpool, Health Services Research<br>Hepworth, Lauren; University of Liverpool, Health Services Research<br>Lowers, Victoria; University of Liverpool, Health Services Research, Institute of Psychology, Health and Society<br>Joseph, Frank; Countess of Chester Hospital<br>Davies, Elizabeth; Royal College of Physicians<br>Gabbay, Mark
**Primary Subject Heading**:	Patient-centred medicine
Secondary Subject Heading:	Sociology
Keywords:	public involvement, Quality improvement, Royal College of Physicians, Future Hospital Programme, Theoretical evaluation, co-production

SCHOLARONE™
Manuscripts

The role of public involvement in the Royal College of Physicians' Future Hospitals healthcare improvement programme: an evaluation

Lucy Frith[1], Lauren Hepworth[1], Victoria Lowers[1], Frank Joseph[2], Elizabeth Davies[2], Mark Gabbay[1]

[1] University of Liverpool, Liverpool, UK

[2] Royal College of Physicians, London, UK

Address for correspondence:

Dr Lucy Frith

Department of Health Services Research

Waterhouse Building, Block B, First Floor,

University of Liverpool,

1-5 Brownlow Street,

Liverpool, L69 3GL

E: l.j.frith@liverpool.ac.uk

T: 0151 7955333

Word Count: 4,512

Key words: Patient public involvement, co-production, co-design, Future Hospital Programme, quality improvement, healthcare improvement, theoretical evaluation

For peer review only - http://bmjopen.bmj.com/site/about/guidelines.xhtml

Abstract

Objectives

The Royal College of Physician's Future Hospital Programme (FHP) set out a blueprint for a radical new model of care that put patient experience centre stage. This paper reports on the results of an independent evaluation of the FHP and focusses on the role public patient involvement (PPI) played in these projects. The paper explores the perceptions and experiences of all those involved in the FHP of how PPI was operationalised in this context; and develops an 'ex-post' programme theory of PPI in the FHP. We conclude by assessing the benefits and challenges of this work.

Setting

Secondary care. The FHP consisted of eight clinician-led healthcare improvement (HCI) hospital development sites with two phases.

Participants

Development site clinical teams, patient representatives, the RCP's Patient and Carer Network, members of the FHP team and fellows and members of the RCP.

Design/Methods

We conducted an independent evaluation of the FHP using FHP documentation, and data collected specifically for the evaluation: qualitative interviews, focus groups and a web-based survey.

Results

The PPI initiatives set out to develop more patient-centred care and improve the patient experience. The mechanisms designed to meet these goals were: 1) A programme of PPI in the development site's projects; 2) A better understanding of patient experience; and 3) Evaluation of patient experience.

Conclusion

This evaluation of the FHP identifies some key elements that need to be considered when attempting to more closely integrate PPI and co-production in service re-design. The structure

For peer review only - http://bmjopen.bmj.com/site/about/guidelines.xhtml

of FHP over two phases enabled learning from Phase I to be incorporated into Phase II. Having the PPI representatives closely involved; developing communities of practice; and the oversight and measuring activities, acted as 'disciplinary structures' that contributed to embedding PPI in the FHP and kept the patient experience at the forefront of the improvement initiatives.

Strengths and limitations of this study

- Contributes to theoretical developments in public involvement as a quality improvement measure.
- Develops an 'ex-post' theory of how public involvement can be used to improve front-line services.
- This evaluation was unable to get robust data on the effects of the public involvement activities on the development site projects.
- Points to areas for future development such as developing mechanisms for measuring the impact of public involvement on service improvement initiatives.

Background

The Future Hospital Programme (FHP) arose out of the Future Hospital Commission (FHC), established by the Royal College of Physicians (RCP) in 2012. The FHC was set up in a response to the RCP's *Hospitals on the Edge Report*[1], that found: 'A health system ill-equipped to cope with the needs of an aging population with increasingly complex clinical, care and support needs; hospitals struggling to cope with an increase in clinical demand; and a systematic failure to deliver coordinated, patient-centred care'[1]. The FHC aimed to, 'set out a radical new model of care' that put patient experience centre stage, 'Patient experience must be valued as much as clinical effectiveness, and patients must be involved in service design and delivery'[2]. A key part of the FHC was to foster and promote patient-centred care, which they defined as, 'individualised, compassionate, holistic and collaborative… [and] continuous,

For peer review only - http://bmjopen.bmj.com/site/about/guidelines.xhtml

care in settings appropriate to the patient's clinical and care needs viewed from their perspective.'[2] While there is debate in the literature over how this concept is and should be defined,[3] key elements of patient-centred care for the FHC were: continuity of care, reduction in unnecessary moving of patients between wards and better integration with social care services post-discharge.

To take the recommendations and vision of the FHC into practice, the RCP set up the FHP in 2013, that included two phases that ran for three and one year respectively. The programme concluded in 2017, however, the sites continued to work on their quality improvement initiatives beyond the end of the FHP. The RCP advertised for clinician-led healthcare improvement (HCI) proposals to become FHP development sites. The first phase was recruited in 2014 and focussed on improving care for older and frail patients. The second phase was a targeted call on integrated care and began in 2016. Each phase included four pilot project sites, all had to align their objectives with the Future Hospital Commission principles (full details of the FHP have been published elsewhere[4 5]). Applicants were required to:

- 'provide details of their projects and how these aligned with the principles of the FHC;
- demonstrate involvement of patient representatives in design and implementation;
- have a local, board level executive sponsor'[4].

The FHP was a multi-faceted programme of work that acted as a support mechanism for the sites to improve front-line services, but it did not provide any additional 'transformational' funding. There were four main ways that the FHP supported HCI in the development sites:

Public patient involvement (PPI) - one of the main elements of the FHP was to foster and support PPI in the development sites HCI projects working closely with the RCP Patient Carer Network (PCN) (established in 2004 to give greater patient and carer input into the work of the RCP). The aim was to have the patient perspective present throughout the projects and

For peer review only - http://bmjopen.bmj.com/site/about/guidelines.xhtml

involve patient representatives actively in the co-production of the project design and implementation.

FHP support structures – the RCP established a FHP team with a senior physician appointed as Future Hospital Officer for each phase who provided clinical leadership support. Collaborative learning structures were developed, such as: learning events where site teams met and shared experiences; training in improvement methodologies; peer support; and creating a community of interest (the Future Hospital Partners Network)[4]. The RCP's PCN also provided support to local lay representatives and peer learning opportunities across all sites.

Reporting processes – development sites were required to report monthly to the FHP team (both written reports and telephone calls). This ensured that the sites maintained momentum and received regular feedback.

Data collection and analysis – sites were given training sessions provided by the RCP or external experts on data analysis, project management and support for collecting and interpreting performance metrics and patient experience data. There was a, 'focus on measuring the true impact of clinically-led improvement or change…[to] enable clinical teams to improve patient-centred care and outcomes'[4].

This paper reports the results of an independent evaluation of the FHP and focusses on the role public patient involvement (PPI) played. The aims of the paper are two-fold: to explore the perceptions and experiences of the central FHP team, the patient representatives and the development site teams of how PPI was operationalised in this context; and to develop an 'ex-post' programme theory of what mechanisms the FHP used to meet its goals of developing

For peer review only - http://bmjopen.bmj.com/site/about/guidelines.xhtml

more patient-centred care and consequently improving the patient experience. We will conclude by assessing the benefits and challenges of this work.

The terms patient public involvement and co-production are contested. Tritter defines PPI as: 'Ways in which patients can draw on their experience and contributors of the public can apply their priorities to the evaluation, development, organization and delivery of health services.'[6] When defining the co-production it is important to recognise that, in the messy realties of practice, how patients and the public participated in the FHP did not always conform to one definitional model, but often included and straddled different types of practice: from passive involvement, such as being consulted about planned changes; to more active involvement, such as being involved in planning; and, in some cases, co-production of initiatives[4 5]. Co-production was used in FHP to mean, 'equal partnerships between patients and physicians in the design of health services.'[4] Osborne et al.[7] define co-production as 'the voluntary or involuntary involvement of public service users in any of the design, management, delivery and/or evaluation of public services'. As has been noted the concept of co-production is very broad and specifically, for the FHP, the key element of co-production was service co-design[8], and we shall use the term co-production/design to denote the form co-production took in the FHP. Definitions of healthcare improvement are also contested[9]. We will define HCI broadly as: 'better patient experience and outcomes achieved through changing provider behaviour and organisation through using a systematic change method and strategies.'[10]

There is a growing literature on the role of PPI in HCI[6-8], but this remains underexplored compared to the use of PPI in other areas, and this paper contributes to this developing area. There is a large literature on theoretical considerations of topologies of involvement and a number of attempts to develop categorisations to capture what PPI is[6 11 12] and what an 'ideal type' might be[13]. Arnsteins' ladder of engagement is often quoted as one of the first examples of this, where the bottom rung is manipulation and the top rung is citizens' power[14]. This paper does not attempt to compare or measure PPI in the FHP according to abstract standards, but

For peer review only - http://bmjopen.bmj.com/site/about/guidelines.xhtml

contributes to the empirical literature on the practice of PPI in service design and quality improvement. It has been noted that although there is a substantial body of theoretical literature, there is a gap on *how* involvement and co-production/design is carried out in practice[15]. It is widely recognised that the context of interventions are key[16] and this paper provides an example of a specific programme of PPI to add to the literature on exploring contextual factors and developing good practice in the area of PPI in HCI.

METHODS

This paper is based on a summative evaluation[17] our team conducted of the FHP as a programme of HCI. It does not consider the specific site improvement projects nor content of these changes (these are reported elsewhere)[4, 18-25]. This paper reports on the perceptions and experiences of the central FHP team, the patient representatives and the development site teams, of how PPI was operationalised in this context and the benefits and challenges of this work. Our team became involved towards the end of the programme and we did not have input in the design of the projects, nor the procedures, content and timing for gathering metrics, and the challenges in doing this kind of research have been discussed in the literature[26]. Thus, this is a consideration of what happened in a HCI initiative in the real world, not one that took place as part of a structured research project. There are a number of strengths and limitations with using evaluations as research data. The strengths are that it provides information on what actually happened in practice, it demonstrates the workings of PPI embedded in the context of shifting priorities and changes in the NHS, such as funding and personnel changes. The main weakness is that from the data collected by the FHP and our evaluation it was difficult to ascertain the effects of the PPI initiatives on the individual development site projects.

We used a multi-source approach using a variety of data sources. We analysed data the FHP collected, reviewing four hundred and seventy-one documents from the development sites, including: monthly, quarterly and annual reports, patient reports, learning event presentations,

For peer review only

posters, feedback, monthly call notes, notes from site visits and personal communications. Members of the team also attended some of the national meetings for the development sites.

We also collected data specifically for the evaluation: focus groups, qualitative interviews and a web-based survey. Focus groups were conducted with all the development site teams, which included the clinical lead and at least one patient representative. A further focus group was conducted with seven local patient representatives and two PCN representatives, in which seven of the eight development sites were represented. One-to-one interviews were offered to all local patient representatives, four were conducted via telephone and one patient representative provided a written account. One-to-one interviews were conducted with key personnel in the RCP, 17 individuals either face to face or via telephone, six from within the FHP core team and 11 who were indirectly involved with the FHP, including three senior members (see table 1 for an overview).

A web-based questionnaire was circulated to the development site team members including the patient representatives after the focus groups, providing another opportunity for team members to share their opinions individually. The survey used a combination of multiple choice and free text response options, with questions based on the topic guide for the focus groups. The survey received 22 responses; this included at least one response from each of the eight development sites and from a mixture of patient representatives and clinicians (for details see[5]). The data and analysis is available in the evaluation report[5], evaluations of the individual site projects[18-25] and the final FHP Report[4].

For peer review only - http://bmjopen.bmj.com/site/about/guidelines.xhtml

Table 1: Overview of qualitative data

	Number	Participants
**Focus group**	1	9 (7 – site patient representatives, 2 – PCN members)
	8	With each development site team<br>Between two and five team members.<br>All groups included the clinical lead and 5 out of the 8 groups included at least one patient representative.
**Interviews**	5	Patient representatives
	6	FHP core team
	11	Members of RCP (including 3 senior members)

All focus groups and interviews were recorded with consent and transcribed verbatim and a constant comparative thematic analysis was used to code the data[27]. Descriptive quantitative statistics were used to analyse the multiple-choice responses of the survey. To maintain participants' anonymity, development sites are referred to as DS1 to DS8, patient representatives as PR1 to PR6 and the personnel in the Future Hospital Programme team as RCP1 to RCP17 (see Table 2 for information on the participants who we quoted). Ethical approval was given by the evaluation team's Institution. The manuscript was prepared using the Standards for Quality Improvement Reporting Excellence (SQUIRE 2.0)[28].

Table 2: Participant information for those quoted in the paper

ID	Phase	Site	Role
PR1	1	5	Patient representative
PR2	1	5	Patient representative
PR3	1	4	Patient representative
PR4	1	3	Patient representative
PR5	2	6	Patient representative
PR6	2	7	Patient representative
DS3	1	3	Clinician
DS8	2	8	Clinician
RCP1	-	-	FHP Team
RCP4	-	-	FHP Team

For peer review only

RCP5 -	-	Other RCP employee
RCP6 -	-	FHP Team
RCP12 -	-	FHP Team

RESULTS

The FHC saw ‘patient experience as important as clinical effectiveness’ and that patient-centred care should drive the improvement of services.[2] This commitment underpinned the FHP’s attempt to re-orientate hospital provision to be more responsive to patients’ needs rather than organised around professional and organisational imperatives.[4] The FHP aimed to do this by not only consulting with patients and the public but initiating processes and structures to facilitate greater PPI and co-production/design of initiatives in the development sites HCI projects.

As has often been noted in the literature[29], HCI programmes seldom start with an explicit programme theory, defined as, ‘an explanation of why the effects observed in a program are likely to have occurred’[30]. Simply describing interventions does not always ensure that they can be replicated or scaled up in other contexts. How certain outcomes come about, the underpinning social processes and the mechanisms that produce these outcomes need theoretical interpretation to aid replication of the intervention[31]. Developing theory can also form the basis of ‘theory orientated evaluation’, that aims to identify ‘the rationale and assumptions about mechanisms that link programs’ processes and inputs to outcomes (both intended and unintended)’[30]. In order to conduct a form of theory-orientated evaluation, we developed a programme theory of PPI as a HCI intervention in the FHP. As the FHP did not set out an explicit programme theory at the outset, we constructed a form of ‘ex post theory’’[30]. We developed this ‘ex-post’ programme theory from our analysis of the data we gathered as part of the evaluation and the wider FHP documentation.

For peer review only - http://bmjopen.bmj.com/site/about/guidelines.xhtml

The main goals of the PPI procedures in the FHP were to develop more patient-centred care and consequently improve the patient experience. The mechanisms designed to meet these goals can be broken down into three elements:

1. Greater PPI and co-production/design of the initiatives – having PPI representatives closely involved in the development site teams will keep them focussed on developing patient-centred care and improving the patient experience, so that this is less likely to be overlooked or marginalised.
2. A better understanding of patient experience – organisations and professionals may not know what constitutes ‘good patient experience’. PPI can provide important, hitherto, missing information and perspectives on patient experience that can be fed into service redesign alongside operational and clinical information.
3. Evaluating patient experience – using patient experience data as a key metric for evaluating quality of care will re-orientate clinical teams to the importance of patient experience and aid the prioritisation of patient-centred care.

How these mechanisms were operationalised in practice and determining the successful aspects of the FHP, are important for thinking through how PPI can be used in service change and improvement and what lessons can be learned for future HCI projects. We have presented the results to address each of these three mechanisms.

PPI and co-production/design of initiatives

Having PPI representatives closely involved in the development site teams was seen as a mechanism to begin to involve patients and move towards greater co-production/design of services to ensure the continual prioritisation of patient-centred care. PPI was implemented from the start of the FHP. The RCP’s PCN was involved in the FHC and setting up the FHP. Each development site had at least one local PPI representative within their team and in Phase II, sites had to show that they had a PPI strategy in place before being chosen. The PPI representatives were meant to be an integral part of the FHP team and be active participants

For peer review only

in team meetings and decision-making processes. The majority (63.6%) of respondents to the development site web-based survey reported that their project was partially co-produced/designed with patients and 27.3% reported that their projects fully involved patients. Co-production/design was limited in some sites, particularly in Phase I, where some projects were already fairly well-developed before any PPI took place.

Although in each Phase the development sites had the same remit and operating instructions it was clear that PPI did not happen in the same way and to the same degree in each site. Comparing the evidence across site reports and phases, there were different levels of involvement and the timings and format of PPI varied. In some instances the involvement was reported to be rather 'tokenistic' and the patient representative's chance to influence service redesign was seen as limited (Table 3, quote 1).

In some of the sites, the PPI representative had little input into setting up the project or its design, but were more involved at later stages. Some participants felt they were brought in after the main decisions were taken (Table 3, quote 2 and 3). However, in some sites patient representatives were more actively involved in setting up and co-designing the projects from their inception (Table 3, quote 4).

The FHP central team reported that from their perspective, PPI was well embedded in the FHP and this enabled more co-production/design of projects (Table 3, quote 5).

Table 3: Levels of involvement and co-production/design of initiatives

Involvement and co-production/design of initiatives	
Quote 1	"Well I have done very little. I have, I haven't had no idea what the patient rep was supposed to do. In my opinion I was merely a tick in a box that said you have to have a patient rep." (PR4)
Quote 2	"It quite frankly is that you don't start off with asking the patients what they want, you start off usually with some enthusiastic usually a clinician, who has an idea about how things might be done better….

For peer review only - http://bmjopen.bmj.com/site/about/guidelines.xhtml

	and the patients are asked to contribute to the development of that idea." (PR5)
Quote 3	"All you ever do is ask them to review what you have done rather than to input into it and you know there are these things where you go, hmm, this is not a co-production the patient is not at the heart of the process of the project." (PR6)
Quote 4	"[I was] very involved. We meet monthly with the … team which is an opportunity to share and discuss ongoing proposals and ideas or implementation of new approaches to working….The patient reps are treated with courtesy and respect and views are listened to and taken on board. We are considered to be an integral and vitally important part of the team." (PR2)
Quote 5	"unique to Future Hospital, in comparison to the other programmes of work. So there is lots of 'oh a patient was involved' tick type activity, throughout the College [RCP], and I think the difference particularly with the phase two sites is that there is proper co-production with the patients I hope that they feel that way, it certainly seems at least a big step along the route to co-production, than anything else that I have been involved with or seen or heard about so far." (RCP1)

A better understanding of patient experience

The FHP sought to get a better understanding of patient experience in two main ways. First, the involvement of the PPI representatives in the site teams and second, the capture of wider patient experience data in each development site. These will be discussed in turn.

The involvement of the PPI representatives in the site teams were seen as important by members of the central FHP team as a way of keeping the focus of the development site teams on patient experience (Table 4, quote 1).

However, the role of the PPI representative, what it should be and who was an appropriate person to occupy this type of role was debated by all FHP participants and there was little consensus on this. The key area of debate was who could be said to accurately provide insights into the patient experience, how do we know we are not just getting a partial view, or views that may not be held by the majority of patients? As one site noted: 'those that are able to meaningfully contribute to system redesign are not necessarily representative of the wider population'[4]. Underpinning these concerns was ambiguity over the concept and purpose of

For peer review only

For peer review only - http://bmjopen.bmj.com/site/about/guidelines.xhtml

being a ‘representative’ and what ‘representativeness’ meant in practice (Table 4, quote 2 & 3).

Table 4: A better understanding of patient experience

A better understanding of patient experience	
Quote 1	“It has helped to keep the clinicians grounded, it has helped to keep the focus on patient experience.” (RCP12)
Quote 2	“I am very much of the opinion that individual patients cannot represent patients as a whole unless it is very strange or peculiar circumstances. Probably, leaders of some patient organisation or something but even then it is a pretty poor sample.” (PR5)
Quote 3	“There is still quite a lot of uncertainty about what your [PPI representative] role is. Are you giving a viewpoint as a patient who has experienced that service, so if you like common sense from an individual point of view or are you in a representative role are you trying to reflect a broader view of patients let's say who are acutely ill going in through a particular hospital. And, that I think hasn’t been worked out nationally we haven’t really got a sort of sense of what the, what the major aspects of a patient representative role are.” (RCP12)
Quote 4	“I think that the disappointing thing is that one considers patient experience, to be reflected by a patient representative. Because patient experience is so much more than just one person coming in and saying…I think the word representative is a very difficult word because I am not sure that [our patient representative] could truly represent patients other than having been one…we have 70,000 patients a year, so the question in my mind is, you know if we are trying to extrapolate a representation of their experience, then having one person who has their own carried experience representing them is difficult.” (DS3)
Quote 5	“If you have a patient representative the… are they a representative of the wider population or are they just bringing their own baggage to it, that is a big question and it is a big, you almost have to train people not to bring their baggage to the table and that is not easy.” (RCP4)
Quote 6	“Patients will speak more openly with us [PPI representatives] than perhaps they feel they can do with the medical or other members of the team.” (PR2) and without their input, “I don’t believe their true voice would have been heard. And so some assumptions would have been made as to what the patient needs.” (PR2)

A clinician in a development site, while supportive of having patients involved, questioned the utility of having a specific PPI representative, as they felt this just reflected one individual’s opinion (Table 4, quote 4). There was a concern over the lack of capacity in PPI and one member of the central FHP team felt that training was needed to enable PPI representatives

For peer review only - http://bmjopen.bmj.com/site/about/guidelines.xhtml

to step outside of their own concerns and be able to represent the wider group (Table 4, quote 5).

A second way of incorporating data on patient experience in the FHP was the systematic capture of patient experience data in each development site.[30] Local patient and PCN representatives played a key role in organising and developing patient experience surveys and other types of data gathering such as running focus groups or public events. The PPI representatives were involved in recruiting, training and supporting volunteers to pilot the patient experience surveys, provide feedback, propose improvements and develop site-specific tailored questionnaires[32]. These were seen as one of the successes of the FHP, that they were able to get the feedback from the wider body of patients and in a more sustained and nuanced way. One development site patient representative spoke about how they were able to get much richer and valuable data on patient experience and what patients wanted from services (see Table 4, quote 6).

Metrics on patient experience

An important part of the FHP was building capacity in data collection and its management so that they could demonstrate the impact of their service changes. One of the key elements of this was developing expertise in capturing, measuring and interpreting patient experience data. 'This metrics orientation provides focus and develops a language of 'measures' that 'quantifies' patient care'[4]. It was seen as important that the metrics captured reflected what was important to patients[4]. The role of this continual data collection in prioritising PPI was seen as crucial to the success of instituting PPI in the FHP (Table 5, quote 1).

For peer review only - http://bmjopen.bmj.com/site/about/guidelines.xhtml

Table 5: Metrics on patient experience

Metrics on patient experience	
Quote 1	"…the patient engagement piece is quite labour intensive, and whether without the Future Hospital Programme guiding it … will providers prioritise it [patient engagement] in the same way they would without the Future Hospital chasing them and asking them what they are doing on a regular basis...". (DS8)
Quote 2	"For phase two having watched how the sort of organic coming together, people volunteering option in phase one for patient reps, hadn't really been as effective as I think we initially hoped and that, the rigours of doing FHP alongside the day jobs…. meant that patient recruitment was often low down on their list and sometimes it took them, a good few months to recruit a patient rep…. So for phase two we decided to do a more proactive recruitment campaign advertised….. So we learnt a lot from phase one, about how to take patient involvement and engagement in the development site teams from sort of tokenistic and leaving the teams to do it themselves, to really prescribing what we needed and to get the framework in place to." (RCP6)

One of the benefits of this continual reporting and assessment of progress and the incorporation of a form of the 'Plan-Do-Study-Act' (PDSA) cycle[33], was that the FHP's approach to PPI evolved between the two phases. This was an iterative process where lessons were learnt and improvements made as the programme progressed. Due to problems experienced in Phase I, where some sites lacked any patient representation for periods of time or had recruited individuals who had difficulties in contributing to the role, changes were made to the application process to be a development site. In Phase II, 'Patient representatives were identified in the application and were integral to the interview processes'[4]. The PCN became involved in the selection of the sites and the recruitment for local site PPI representatives became more formalised. The development of the PPI representatives' role and the overall strategy and implementation over the two phases of the FHP is outlined by a member of the FHP team (see Table 5, quote 2). Other improvements included providing more support for the development site PPI representatives. For example, they were given a buddy from the central PCN and this relationship became more formalised in Phase II.

For peer review only

For peer review only - http://bmjopen.bmj.com/site/about/guidelines.xhtml

DISCUSSION

The FHP commitment to involving local patients, PPI representatives and PCN members in service design, monitoring and evaluation was a new experience for the RCP and the PCN[32]. It was recognised by all parties that 'doing' PPI was challenging and required significant commitment on the part of all members of the development site teams.

The lack of theoretical consideration of how PPI might contribute to service change and create the conditions for patient-centred care can make evaluation challenging, and the paucity of underpinning theory in this area has been noted in the literature[29 30]. In order to design, carry out and continuously evaluate PPI in HCI projects, having a working programme theory can provide clear aims and objectives against which to assess whether the PPI mechanisms have fulfilled their stated aims and if, and how, PPI as an intervention has worked. How such theoretical underpinnings of PPI as an intervention can and should be developed, are areas that needs further investigation. We developed an 'ex-post programme theory' of PPI as an HCI intervention and setting out the goals for this kind of programme is a first step to developing ways of assessing these initiatives. Our study has shown that more work needs to be done on developing appropriate metrics for assessing PPI as a HCI tool.

The initial requirements for sites to have a PPI strategy, a patient representative and good links with the PCN were important framing mechanisms for the project. This kept the focus on patient experience and it was by being a FHP development site that this was promoted and sustained. As site members observed, along with all the other requirements of both the HCI projects and day-to-day business, it would have been easy for PPI to get subsumed. The communities of learning that the FHP support structures created and attending learning events with other sites[4] acted as a disciplinary structure, a community of practice that kept PPI at the forefront of the projects[30]. The support and endorsement for PPI from a prestigious medical college was also a key factor in PPI being seen as an important and worthwhile activity. This way of improving practice through professional communities and expert reassurance has been

For peer review only - http://bmjopen.bmj.com/site/about/guidelines.xhtml

used in other studies[34], and worked well in the FHP, although other studies suggests more mixed evidence of their effectiveness[35].

A challenge with getting comprehensive information on patient experience is that only a few patients were and could be involved in the PPI initiatives, and therefore, this information could be argued to only partially capture the patient experience. Even when sites tried to organise public involvement days, these often did not attract much interest from patients. Further, concepts of how patient representatives 'represent' are contested in PPI[36]. Several sites saw the role of the patient representatives as a 'patient voice' rather than providing a representative account of 'patient experience'. Therefore, the ability to attract a range of patients' views and get wider understanding of patient experience was often limited. However, sites generally saw the value of including patient representatives in clinical teams and reported that they found the contribution of patients extremely valuable. Several sites were aiming to increase the level of patient input, to help them response to the high levels of changing demand among their population groups. Thus, adding extra in-house capacity and developing arrangements for closer working with patient groups was seen as a high priority for future service improvement. The work done by the patient representatives: running patient surveys, developing and piloting new iterations and exploring qualitative data gathering mechanisms, such as interviews and focus groups, helped produce much richer information and site teams used this to both monitor their progress and feed into further improvement cycles.

Finally, the FHP was designed to enable sites to instigate robust systems for measuring progress and giving the sites the skills to set up data gathering structures. 'Measurement always has consequences'[30] and by having patient experience data as a key performance indicator and the requirement to report to the central FHP team regularly, it ensured both that this remained an important focus for the projects, and that the patient representatives had a clearly defined and important role.

For peer review only - http://bmjopen.bmj.com/site/about/guidelines.xhtml

Learning between Phases

A strength of the FHP was employing learning from Phase I into Phase II and embedding a PDSA cycle in the programme. This meant key changes were made in Phase II that strengthened PPI: involving the PCN in the process of choosing sites, strengthening processes for recruiting patient representatives, formalising the buddy system between the local representatives and a PCN member; and recommending each team have two patient representatives to share the work and provide support[32].

Limitations

This was an evaluation[5] of an existing and ongoing project and the data and measurement processes were not determined by the evaluation team. Thus, this evaluation is based on the available data and resources. The FHP did not include an impact assessment of the PPI activity itself and hence, we were not able to get detailed process or quantitative data on the activities undertaken by either the local or PCN patient representatives[5]. As has been noted, assessing the impact of PPI on HCI projects is challenging, and embedding key measures of success throughout the project would have provided a useful way of assessing progress during the FHP. The lack of studies on how PPI effects the outcomes of service reconfigurations has been noted in Dalton et al's review[37], and ways to measure the impact of PPI are generally under-researched.[38] This is an area that needs further research to develop ways of effectively capturing the impact and effects of PPI in service design and change. For future HCI, our study highlighted the need for improvement teams to have a programme theory of how the intervention could work from the offset that includes: clear goals of the initiative; how concepts such as co-production and related concepts such as co-design are defined subsequently employed; what markers of success might look like; and clear mechanisms for achieving this. By having a programme theory to guide setting up the initiative and establishing mechanisms of achieving key outcome measures, it should be possible to determine the impact of PPI in HCI with more accuracy.

For peer review only - http://bmjopen.bmj.com/site/about/guidelines.xhtml

CONCLUSIONS

Developing an ethos of co-production in the form of co-design, even if this was often moving towards rather than fully achieved, was a major success of the FHP. To meaningfully embed PPI in HCI projects it needs to be sustained and sustainable, and requires significant investment in support structures, both centrally and locally, plus time and space allocated to enabling decisions to be genuinely co-produced. There needs the support of the whole system – organisational buy in – so that it becomes a 'way of doing things' throughout the HCI project and once the initiative is rolled out. Additionally, there needs to be proper investment of staff time and associated costs to deliver real PPI and co-production. For example, the project teams need dedicated time for meetings to start the design process, rather than involving patient representatives when the projects are already set up, as this inevitably precludes genuine co-production. Demonstrating the impact of PPI in HCI projects is the next challenge and there is a need to develop methodologies that can capture relevant metrics and the nuances of involvement. Combining qualitative and quantitative data and embedding collection throughout the project can give a more multi-dimensional picture[26]. Attention to developing programme theories of PPI in HCI will be a key step and this paper aimed to contribute to this debate by developing an 'ex-post theory' that could be used to suggest ways of developing PPI as an HCI tool.

Acknowledgements

We would like to acknowledge and express thanks to all the members of the development site teams, the PCN, staff at the Royal College and Physicians and Members and Fellows of the RCP who took the time to speak to us or complete a survey.

The authors would also like to thank the Professor Tom Walley for his support with this work.

Professor Mark Gabbay and Dr Lucy Frith are part-funded by the National Institute for Health Research (NIHR) Collaboration for Leadership in Applied Health Research and Care North

For peer review only - http://bmjopen.bmj.com/site/about/guidelines.xhtml

West Coast (CLAHRC NWC). The views expressed are those of the author(s) and not necessarily those of the NIHR or the Department of Health and Social Care.

Disclaimer

The views expressed here are those of the authors and not necessarily those of the NHS, the NIHR, or the Department of Health and Social Care.

Ethics

University of Liverpool, Psychology Health and Society Ethics Committee, IPHS-1907

Funding statement

This evaluation was funded by the Royal College of Physicians.

Competing interests

We have not competing interests to declare.

Author contribution

LF – analysed the data, drafted the paper, LH, MG, VL – analysed the data, contributed to writing and revising the paper, FJ – contributed to writing and revising the paper, ED – public contributor, contributed to writing and revising the paper.

Data sharing statement

All requests should be directed to the corresponding author.

References

1. Royal College of Physicians. Hospitals on the edge? The time for action. London: RCP, 2012.
2. Future Hospital Commission. Future Hospital: Caring for medical patients. London: Royal College of Physicians, 2013.

For peer review only - http://bmjopen.bmj.com/site/about/guidelines.xhtml

3. Dewing J, McCormack B. Tell me, how do you define person-centredness? *Journal of Clinical Nursing* 2016;26(17-18):2509-10.
4. Future Hospital Programme. Delivering the future hospital: full report London: Royal College of Physicians; 2017 [Available from: www.rcplondon.ac.uk/projects/outputs/future-hospital-programme-delivering-future-hospital.
5. Hepworth L, Antonacopoulou E, Frith L, et al. Future Hospital Programme: an independent evaluation - Final Report: University of Liverpool, 2017.
6. Tritter JQ. Revolution or evolution: the challenges of conceptualizing patient and public involvement in a consumerist world. *Health Expectations* 2009;12(3):275-87.
7. Osborne SP, Radnor Z, Strokosch K. Co-production and the co-creation of value in public services: a suitable case for treatment? *Public Management Review* 2016;18(5):639-53.
8. Loeffler E, Power G, Bovaird T, et al., editors. *Co-production of health and wellbeing in Scotland*. Birmingham, UK: Governance International, 2013.
9. The Health Foundation. Quality improvement made simple: What everyone should know about health care quality improvement. London: The Health Foundation, 2013.
10. Øvretveit J. Does improving quality save money? A review of the evidence of which improvements to quality reduce costs to health service providers. London: The Health Foundation, 2009.
11. Oliver SR, Rees RW, Clarke-Jones L, et al. A multidimensional conceptual framework for analysing public involvement in health services research. *Health Expectations* 2008;11(1):72-84.
12. Rowe G, Frewer LJ. A typology of public engagement mechanisms. *Science, Technology and Human Values* 2005;30(2):251-90.
13. Fredriksson M, Tritter JQ. Disentangling patient and public involvement in healthcare decisions: why the difference matters. *Sociology of Health and Illness* 2017;39(1):95-111.
14. Arnstein SR. A ladder of citizen participation. *Journal of American Planning Association* 1969;35(4):216-24.
15. Ward ME, De Brún A, Beirne D, et al. Using co-design to develop a collective leadership intervention for healthcare teams to improve safety culture. *International Journal of Environmental Research and Public Health* 2018;15(6) doi: 10.3390/ijerph15061182
16. De Weger E, Van Vooren N, Luijkx KG, et al. Achieving successful community engagement: a rapid realist review. *BMC Health Services Research* 2018;18(285) doi: 10.1186/s12913-018-3090-1
17. The Health Foundation. Evaluation: what to consider. Commonly asked questions about how to approach evaluation of quality improvement in health care. London: The Health Foundation, 2015.
18. Williams O, Betsi Cadwaladr University Health Board Future Hospital Development Site Team. Phase 1 Future Hospital Development Site: Betsi Cadwaladr University Health Board Final Report. London: Royal College of Physicians, 2017.
19. East Lancashire Hospitals NHS Trust Future Hospital Development Site Team. Phase 1 Future Hospital Development Site: East Lancashire Hospitals NHS Trust Final Report. London: Royal College of Physicians, 2017.
20. Duckitt R, Hunt D, Finch L, et al. Phase 1 Future Hospital Development Site: Worthing Hospital Final Report. London: Royal College of Physicians, 2017.
21. Mid Yorkshire Hospitals NHS Trust Future Hospital Development Site Team. Phase 1 Future Hospital Development Site: Mid Yorkshire NHS Trust Final Report. London: Royal College of Physicians, 2017.
22. Kane B, O'Reilly S, Noble R, et al. Phase 2 Future Hospital Development Site: Central and South Manchester Final Report. London: RCP, 2017.
23. Arkwright P, Dixon S, Stafford N. Phase 2 Future Hospital Development Site: North West Paediatric Allergy Network. London: Royal College of Physicians, 2017.
24. Rajasakeran A, Barlow R, Chadderton M, et al. Phase 2 Future Hospital Development Site: Sandwell and West Birmingham Final Report. London: Royal College of Physicians, 2017.

For peer review only

For peer review only - http://bmjopen.bmj.com/site/about/guidelines.xhtml

25. Compton L, Lawn L, Wilkinson P, et al. Phase 2 Future Hospital Development Site: North West Surrey Final Report. London: Royal College of Physicians, 2017.
26. Raine R, Fitzpatrick R, Barratt H, et al. Challenges, solutions and future directions in the evaluation of service innovations in health care and public health. *4* 2016;16
27. Braun V, Clarke V. Using thematic analysis in psychology. *Qualitative Research in Psychology* 2006;3(2):77-101.
28. Ogrinc G, Davies L, Goodman D, et al. SQUIRE 2.0 (Standards for QUality Improvment Reporting Excellence) revised publications guidelines from a detailed consesnus process. *BMJ Quality and Safety* 2016;25(12) doi: 10.1136/bmjqs-2015-004411
29. Davidoff F, Dixon-Woods M, Leviton L, et al. Demystifying theory and its use in improvement. *BMJ Quality and Safety* 2015;24:228-38.
30. Dixon-Woods M, Bosk CL, Aveling EL, et al. Explaining Michigan: Developing an ex post theory of a quality improvement program. *Milbank Quarterly* 2011;89(2):167-205.
31. Howarth E, Devers K, Moore G, et al. Contextual issues and qualitative research. In Raine R, Fitzpatrick R, Barratt H, Bevan G, Black N, Boaden R, *et al.* Challenges, solutions and future directions in the evaluation of service innovations in health care and public health. *Health Services Delivery Research* 2016;4(16):105-20.
32. Caporn CA, Sawicka ZS. Making ripples: patient involvement in service planning. *Future Hospital Journal* 2016;3(2):142-46.
33. Institute for Healthcare Improvement. QI Essentials Toolkit: Plan-Do-Study-Act worksheet Cambridge, Massachusetts, USA Institute for Healthcare Improvement; 2017 [Available from: www.ihi.org/resources/Pages/Tools/PlanDoStudyActWorksheet.aspx.
34. Jeffs L, McShane J, Flintoft V, et al. Contextualizing learning to improve care using collaborative communities of practices. *BMC Health Services Research* 2016;16(464) doi: 10.1186/s12913-016-1566-4
35. Aveling E-L, Martin G, Armstrong N, et al. Quality improvement through clinical communities: Eight lessons for practice. *Journal of Health Organisation and Management* 2012;26(2):158-74.
36. Maguire K, Britten N. "How can anybody be representative for those kind of people?" Forms of patient representation in health research, and why it is always contestable. *Social Science and Medicine* 2017;183:62-69.
37. Dalton J, Chambers D, Harden M, et al. Service user engagement and health service reconfiguration: a rapid evidence synthesis. *Health Services Delivery Research* 2015;3(17) doi: 10.3310/hsdr03170
38. Mockford C, Staniszewska S, Griffiths F, et al. The impact of patient and public involvement on UK NHS health care: a systematic review. *International Journal for Quality in Health Care* 2012;24(1):28-38.

For peer review only - http://bmjopen.bmj.com/site/about/guidelines.xhtml

Research and reporting methodology
Revised **Standards for QUality Improvement Reporting Excellence** (**SQUIRE 2.0**) publication guidelines

Notes to authors

- The SQUIRE guidelines provide a framework for reporting new knowledge about how to improve healthcare.
- The SQUIRE guidelines are intended for reports that describe system level work to improve the quality, safety and value of healthcare, and used methods to establish that observed outcomes were due to the intervention(s).
- A range of approaches exists for improving healthcare. SQUIRE may be adapted for reporting any of these.
- Authors should consider every SQUIRE item, but it may be inappropriate or unnecessary to include every SQUIRE element in a particular manuscript.
- The SQUIRE glossary contains definitions of many of the key words in SQUIRE.
- The explanation and elaboration document provides specific examples of well-written SQUIRE items and an in-depth explanation of each item.
- Please cite SQUIRE when it is used to write a manuscript.

Text section and item name	Page/line no(s). info is located
**Title and abstract**	
1. **Title**	
Indicate that the manuscript concerns an initiative to improve healthcare (broadly defined to include the quality, safety, effectiveness, patient-centredness, timeliness, cost, efficiency and equity of healthcare).	Line 1
2. **Abstract**	
a. Provide adequate information to aid in searching and indexing.	Lines 4-24
b. Summarise all key information from various sections of the text using the abstract format of the intended publication or a structured summary such as: background, local problem, methods, interventions, results, conclusions.	Page 1, lines 4-30
**Introduction: Why did you start?**	
3. **Problem description** - Nature and significance of the local problem.	Lines 51-59
4. **Available knowledge** - Summary of what is currently known about the problem, including relevant previous studies.	Lines 103-105<br>Lines 149-154
5. **Rationale** - Informal or formal frameworks, models, concepts and/or theories used to explain the problem, any reasons or assumptions that were used to develop the intervention(s) and reasons why the intervention(s) was expected to work	Lines 69-71<br>Lines 163-172
6. **Specific aims** - Purpose of the project and of this report.	Lines 96-105
**Methods: What did you do?**	
7. **Context** - Contextual elements considered important at the outset of introducing the intervention(s).	Lines 51-71

For peer review only

8. **Intervention(s)**	
a. Description of the intervention(s) in sufficient detail that others could reproduce it.	Lines 71-94
b. Specifics of the team involved in the work.	Line 114 – reference to full evaluation report
9. **Study of the intervention(s)**	
a. Approach chosen for assessing the impact of the intervention(s).	Lines 108-109
b. Approach used to establish whether the observed outcomes were due to the intervention(s).	We were unable to do this due to the evaluation design lines 329-336
10. **Measures**	
a. Measures chosen for studying processes and outcomes of the intervention(s), including rationale for choosing them, their operational definitions and their validity and reliability.	Lines 108-115
b. Description of the approach to the ongoing assessment of contextual elements that contributed to the success, failure, efficiency and cost.	Partly N/A as this was an evaluation of a programme that had partly taken place<br>Lines 117-130
c. Methods employed for assessing completeness and accuracy of data.	Line 114
11. **Analysis**	
a. Qualitative and quantitative methods used to draw inferences from the data.	Lines 108-115
b. Methods for understanding variation within the data, including the effects of time as a variable.	Lines 117-116
12. **Ethical considerations** - Ethical aspects of implementing and studying the intervention(s) and how they were addressed, including, but not limited to, formal ethics review and potential conflict(s) of interest.	Line 134-137
**Results: What did you find?**	
13. **Results**	
a. Initial steps of the intervention(s) and their evolution over time (eg, time-line diagram, flow chart or table), including modifications made to the intervention during the project.	Lines 61-71<br>Lines 251-264
b. Details of the process measures and outcomes.	We were unable to do this due to the evaluation design lines 329-336
c. Contextual elements that interacted with the intervention(s).	Lines 190-195
d. Observed associations between outcomes, interventions and relevant contextual elements.	Lines 243-249
e. Unintended consequences such as unexpected benefits, problems, failures or costs associated with the intervention(s).	Lines 254-264

For peer review only

f. Details about missing data.	Lines 329-336
**Discussion: What does it mean?**	
14. **Summary**	
a. Key findings, including relevance to the rationale and specific aims.	Lines 267-270
b. Particular strengths of the project.	Lines 277-279<br>Lines 279-292<br>Lines 302-311<br>Lines 321-326
15. **Interpretation**	
a. Nature of the association between the intervention(s) and the outcomes.	N/a
b. Comparison of results with findings from other publications.	Lines 287-292<br>Lines 298-299<br>Lines 313-318
c. Impact of the project on people and systems.	Lines 302-311
d. Reasons for any differences between observed and anticipated outcomes, including the influence of context.	N/A
e. Costs and strategic trade-offs, including opportunity costs.	N/A
16. **Limitations**	
a. Limits to the generalisability of the work.	Lines 329-336
b. Factors that might have limited internal validity such as confounding, bias or imprecision in the design, methods, measurement or analysis.	Lines 329-336
c. Efforts made to minimise and adjust for limitations.	N/A
**Conclusions**	
a. Usefulness of the work.	Lines 340-344
b. Sustainability.	Lines 303-307
c. Potential for spread to other contexts.	Lines 303-307
d. Implications for practice and for further study in the field.	Lines 345-346
e. Suggested next steps.	Lines 347-349
**Other information**	
18. **Funding** - Sources of funding that supported this work. Role, if any, of the funding organisation in the design, implementation, interpretation and reporting.	Included in the submission

Ogrinc G, et al. BMJ Qual Saf 2015;0:1–7. doi:10.1136/bmjqs-2015-004411
Downloaded from http://qualitysafety.bmj.com/ on January 2, 2017

For peer review only
